# An In Silico Approach for Assessment of the Membrane Transporter Activities of Phenols: A Case Study Based on Computational Models of Transport Activity for the Transporter Bilitranslocase

**DOI:** 10.3390/molecules24050837

**Published:** 2019-02-27

**Authors:** Katja Venko, Marjana Novič

**Affiliations:** Laboratory for Cheminformatics, Theory Department, National Institute of Chemistry, SI-1000 Ljubljana, Slovenia

**Keywords:** phenols, membrane transporters, bilitranslocase, transport activity, QSAR, in silico models

## Abstract

Phenols are the most abundant naturally accessible antioxidants present in a human normal diet. Since numerous beneficial applications of phenols as preventive agents in various diseases were revealed, the evaluation of phenols bioavailability is of high interest of researchers, consumers and drug manufacturers. The hydrophilic nature of phenols makes a cell membrane penetration difficult, which imply an alternative way of uptake via membrane transporters. However, the structural and functional data of membrane transporters are limited, thus the in silico modelling is really challenging and urgent tool in elucidation of transporter ligands. Focus of this research was a particular transporter bilitranslocase (BTL). BTL has a broad tissue expression (vascular endothelium, absorptive and excretory epithelia) and can transport wide variety of poly-aromatic compounds. With available BTL data (pKi [mmol/L] for 120 organic compounds) a robust and reliable QSAR models for BTL transport activity were developed and extrapolated on 300 phenolic compounds. For all compounds the transporter profiles were assessed and results show that dietary phenols and some drug candidates are likely to interact with BTL. Moreover, synopsis of predictions from BTL models and hits/predictions of 20 transporters from Metrabase and Chembench platforms were revealed. With such joint transporter analyses a new insights for elucidation of BTL functional role were acquired. Regarding limitation of models for virtual profiling of transporter interactions the computational approach reported in this study could be applied for further development of reliable in silico models for any transporter, if in vitro experimental data are available.

## 1. Introduction

Phenols are one of the most promising groups of dietary preventive agents [1,2]. They are widely amenable natural and nutritional agents, i.e. nutraceuticals, which belong to a heterogeneous family of chemical compounds comprising benzoquinones, phenolic acids and aldehydes, coumarins, chromones, xanthonoids, stilbenoids, flavonoids, lignans, tannins, etc. [3]. In general, phenolic compounds are classified based on the number of phenol units in the molecule, as simple phenols or polyphenols [2,4]. Functionally, they are secondary metabolites produced by plants and microorganisms or synthesized industrially [5,6]. As the most abundant antioxidants present in a human normal diet they have received increasing interest from researchers, consumers, food and drug manufacturers [2,7]. The phenols, being recognized as one of the most potential antioxidants, have role in preventing an imbalance in reactive oxygen species (ROS) production and an attenuation of anti-oxidant defense, what can turnout the genesis of many ageing phenomena and diseases [1,8,9,10]. Furthermore, bioactivities of phenols appeared to be quite broad-spanning, yet many multi-step interactions have not been exactly elucidated. Thus, numerous beneficial application of phenols as preventive and therapeutic agents in cancer and inflammatory, neurodegenerative and cardiovascular diseases have been reported [1,9,11,12,13,14,15,16,17,18,19,20,21,22,23,24,25,26,27,28,29,30,31,32]. Particularly, the initiation stages of carcinogenesis were shown to be largely preventable and susceptible to modulation by nutraceuticals, especially highlighting the dietary polyphenols as one of the most promising anti-carcinogenic agents [10,15]. However, solely the normal diet phenolic intake is insufficient for the effective chemoprevention, but the ingestion of extra dietary phenolic supplements as support to chemotherapy is a promising approach [7,10]. The epidemiological data show that life-long intake of phenolic compounds is beneficial and related to reduce the incidence of various diseases [8,11,15,33], yet to improve our knowledge on the bioavailability of polyphenols from the diet uptake is of high interest. 

**Figure 1:**
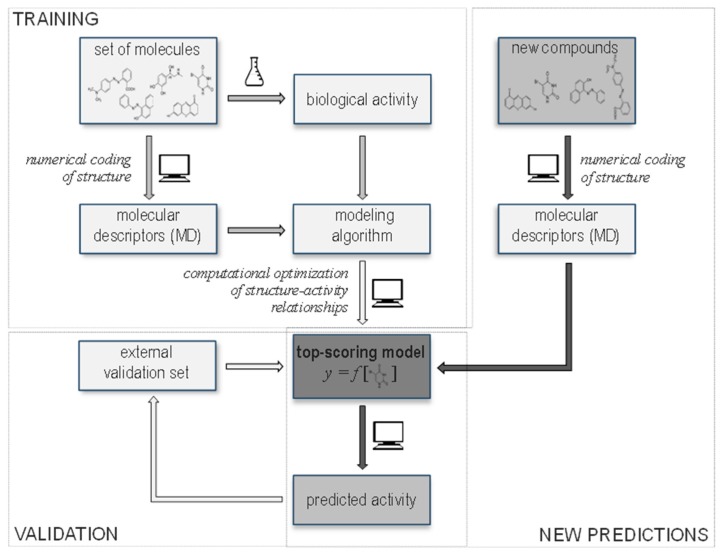
General scheme of the quantitative structure-activity relationship (QSAR) strategy. Chemical structures and biological activity data are obligated input for model building and validation. After the model development and optimization the new activity predictions can be made based solely on chemical structure input.

The uptake of nutraceuticals and orally administrated drugs is dependent on their capacity of absorption from the digestive tract. The studies, which followed the oral ingestion and gastro-intestinal uptake of flavonoids, show that the concentrations are <1% of the administrated dose [34,35]. Such stringent intestinal control can be explained with the experimental evidence that many importers are unable to transport phenols, while various exporters can [34]. This complexity obscures the delivery of phenolic nutraceuticals or drugs, and raises the need for good substrate permeability predictive models. Since phenols are widely distributed in fruits, vegetables, grains and their processed foodstuffs, but are not synthesized by animal cells, their detection in animals is solely indicative of plant ingestion [36]. Therefore, their absorption and tissue distribution in specimens is indispensably dependent on transport ability of membrane transporters, and in this regard the cellular uptake of phenolic compounds can be efficiently estimated by in vitro transport assays [37,38,39], while in silico applications of membrane transport processes are very limited due to extremely slow rise of high-resolution structures of membrane transporters [40]. At present, the development of in silico methodologies for filling the gap among protein structures and properties is emerging and helps circumventing the difficulties in resolving membrane transporter structures and functions [40,41,42]. In general, two in silico strategies exists: (i) ligand-based methods enabling analysis of transporter substrate specificity in the absence of 3D structure, and (ii) protein-based methods providing modeling of transporters structures based on sequence identity of available experimentally determined atomic structures [43]. Among first, the pharmacophore mapping and development of quantitative structure-activity relationship (QSAR) models are the most common for predicting ligand binding modes (Figure 1) [44]. Among second, the comparative modeling is the most frequently used approach to reveal structure and function of transporter, yet is only applicable, if homolog structures are available [45]. Furthermore, studies show that the combination of both strategies is favorable as it provides information beyond individual models [45]. Due to shortage of currently available experimental functional and structural data on membrane transporters, the in silico modelling is essential in the discovery of adverse effects, ADMET properties and more efficient transporter substrates (e.g., dietary products, drugs) [44]. 

By evidence dietary substances and drugs in mammals are transported via membrane transporters rather than passive diffusion [38]. Therefore, it is of interest to profile the membrane transport ability by using the recently available transporter databases in the public domain like Metrabase (www-metrabase.ch.cam.ac.uk) [46] and ChemBench (www.chembench.mml.unc.edu) [44]. Metrabase provides the structural, physiochemical and biological data about transporters and their ligands. It contains 16 efflux and influx transporters, which are also included in the guidance for the approval of drugs proposed by the US Food and Drug Administration and by the European Medicines Agency [46]. On the other hand, Chembench platform offers the largest collection of human intestinal transporters interactions and the compendium of QSAR models for virtual screening of chemicals. Moreover, these two databases reflect the current research focus in mammalian transporters, as up to the best of our knowledge they encompass majority of existing transporters studies. Nevertheless, according to the reports in TransportDB (www.membranetransport.org) [47] and Transporter Classification Database (www.tcdb.org) [48] a hundreds of potential transporters exist (in the human genome > 1400 unique transport proteins were identified). Therefore, our interest arises on a specific, neglected but extremely attractive transporter bilitranslocase (BTL, Uniprot O88750, GenBank: Y12178.1).

**Figure 2:**
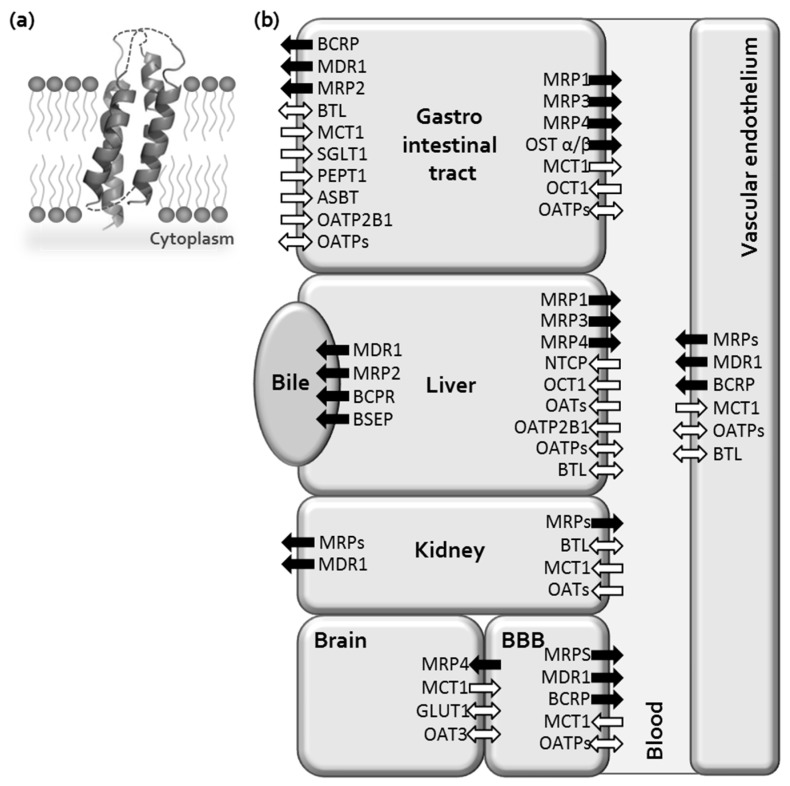
(**a**) Structural model of membrane transporter bilitranslocase (top-scoring computational arrangement of four transmembrane helices based on NMR spectroscopy data) [62]. No homologs were defined for 1026 bp mRNA sequence (GenBank: Y12178.1), thus the comparative modeling studies are not possible to perform. The further structure characterization of bilitranslocase is under study. (**b**) Scheme of cell membrane transporters distribution in different tissues [44,50,58]. ABC transporters are indicated by black arrows and solute carriers (SLC) by white arrows; one direction transport (one way arrow), bidirectional transport (two way arrow).

Bilitranslocase is a 38.22 kDa bilirubin membrane transporter and is the first reported member of the Bilirubin Transporter (BRT) Family (TCDB 2.A.65.1.1) [35]. The gene of BTL is unidentified, and even no homology with any other known protein structure was found [35,49]. Yet, a surprising feature of BTL is its coding sequence, which is 94% homologous to a segment of the antisense strand of the ceruloplasmin transcript (RNO 30106) in the anti-CODE database (www.anticode.org). The considerable biological importance in BTL sequence has the bilirubin-binding motif, which plays a central role in ligand binding, and it is similar to highly conserved segment in α-phycocyanins [49], which are ancient biliproteins present in cyanobacteria. Further on, BTL shares similar tissue distribution and functional properties with some of the members of the family of organic anion transporters (OAT) (Figure 2), which is the most dominant family of the solute carriers (SLC, one of the two classified categories of transporters) [50]. BTL does not transport only bilirubin into hepatocytes [51,52], but can transport a wide variety of poly-aromatic compounds among vitally important endogenous substrates and drugs [35,53,54]. Its tissue expression is broad from vascular endothelium to stomach, intestine, kidney and liver epithelia (Figure 2) [55,56,57,58]. These peculiarities make BTL a hot spot for future investigation as potential important endogenous drug delivery system to the intracellular targets, especially for its ability of cancer drugs uptake [59] and also as tumor biomarker [60]. The study on renal carcinoma cells figure out an outstanding fact, that BTL expression is severely down-regulated in the renal cortical tumors [61], therefore the BTL assessment with specific BTL antibodies could serve as a novel biomarker of human kidney cancers [60]. In spite of our high interest and several efforts, the synthesis of BTL was unsuccessful, thus its structural and functional characterization remains unclear. However, up to date the data on tissue expression based on the specific BTL monoclonal antibodies [35], transport activity assays [53,54] and computational elucidation of its 3D structure based on available NMR data (Figure 2) [62] are available. Since BTL gene is not defined, the BTL characterization experimentally still remains limited only on immunological approach by specific ant-peptide antibodies and in silico modeling with identified synthetic transmembrane segments.

Considering the current limitation and dearth of empirical data about the interesting BTL transporter, the usage of in silico approaches is more than welcome to elucidate the BTL functional properties. Moreover, they can even provide new guidelines for further design of the BTL experiments. Some QSAR models for prediction of the BTL transport ability were preliminary build on available data of biological assay of the BTL transport activity for the poly-aromatic organic compounds [53,54,59]. The in vitro experiments showed fast kinetics of plasma appearance of polyphenols and their concentrations were found in the range of half-saturation of BTL in the portal blood [63]. Thus, Passamonti et al. suggest that particularly at the gastric level the BTL-mediated mechanism could be the basis of the fast kinetics and ability of polyphenols to permeate the gastric mucosa [35,63]. Even more, it seems that the fast absorption of polyphenols in the upper digestive tract is a mindful steep, as polyphenols in the lower segments of digestive tract are exposed to intensive chemical and microbial degradation [1,34], which significantly affect their bioavailability. 

The aim of this study was to assess the membrane transporters activity of selected phenolic compounds by a comprehensive virtual profiling. Firstly, we developed new robust and reliable models for BTL transport activity that were used for screening of new untested dataset of 300 phenolic compounds (including potential drug candidates, nutraceuticals). With the developed QSAR models the new insights into transport interactions of structurally unresolved BTL transmembrane protein were gained and the phenolic compounds that could inhibit the BTL transport were identified. We also discussed the preferences of using the consensus of models to enhance predictivity of in silico models. Secondly, the unique transporter profile of studied compounds was performed based on the results from a new BTL models developed in this study and from the results obtained from Metrabase [46] and Chembench platforms [44], a freely available tools for membrane transporters characterization. 

## 2. Results

### 2.1. BTL Classification Models

The development of QSAR models is a complex process therefore general OECD principles to properly produce and validate models [64,65] have been taken into account. In order to build reliable models and also to detect possible model pathologies in the modelling strategy, a cross validation and external validation was included. Thus, BTL dataset (*n* = 120) was divided into two or three subsets in rate 75/25 or 60/25/15. The model NN-C had three subsets; training set (*n* = 70), test set (*n* = 31) and validation set (*n* = 19). Models NN-D and Q-D had two subsets; training set (*n* = 90) and validation set (*n* = 30). A dataset splitting conditions are precisely stated in study of Martinčič et al. [59].

**Figure 3:**
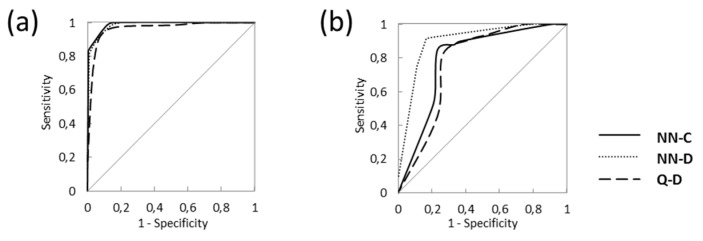
ROC curves of the three selected classification models: (**a**) training set, (**b**) validation set.

Initial modeling datasets included 66 or 78 variables, Codessa and Dragon descriptors, respectively. The model NN-C was the best model available from study of Martinčič et al. [59] and was developed with non-reduced number of descriptors (66 Codessa MDs). In this study new Dragon molecular descriptors (MDs) were calculated and further model optimization with cross validation and genetic algorithm was used. The newly developed models (NN-D and Q-D) contain significantly reduced set of MDs (from 78 to 18/11). The list of selected descriptors of NN-D and Q-D models is represented in Table S4 (Supplementary Material). The selected models have comparable quality parameters for training set, yet new CP-ANN model has significantly improved performance of validation set (Table 1 and Table 2). Regarding results of quantitative quality indicators and graphical quality parameter (ROC curve) the NN-D model shows the best training and validation performances (Figure 3). Predictions for compounds used in the models development and validation are presented in Table S1 (Supplementary Material). 

**Figure 4:**
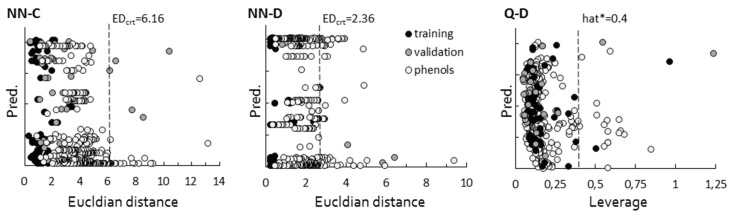
Graphical assessment of the applicability domains of the best three classification models (NN-C, NN-D, Q-D). Compounds with estimated Euclidian distances/leverages > ED_crit_/hat* fail out of applicability domain of model.

The reliability of a QSAR model depends on its capacity to achieve confident predictions of new compounds not considered in the model development, which implies the necessity of considering the applicability domain (AD) of the model. Several AD analyses including the descriptor’s range, leverages, Euclidian distances, rivality and modelability indexes [66,67,68] are available. In this study we used two of AD approaches; maximal Euclidian distance of training set in NN-C (EDcrt = 6.16) and NN-D model (ED_crt_ = 2.36), and leverage values in Q-D model (hat* = 0.4) (Figure 4) [67,68].

The structure activity relationship analysis has revealed the influential structural properties of BTL ligands. In general, high capability of compounds of forming hydrogen bonds, ionic properties and planarity of molecules are the most relevant one. The same features were highlighted in previous BTL experimental studies [53,54,59]. The molecular descriptors, which are used in newly developed models NN-D and Q-D (Table S4, Supplementary Material) represent the following properties: planarity (number of multiple and aromatic bonds — nBM, nArCO, nArOR, ARR), hydrogen bonds forming ability (nOHp, nO, H-052, P_VSA_LogP_2, P_VSA_p_4, CATS2D_07_DA, CATS2D_04_AN), ionic properties (MATS7e, GATS5i, EE_B(s)).

#### 2.1.1. Consensus of Model Predictions

Consensus modeling was performed by integrated predictions among two QSAR models (2-model consensus, three possible combinations) and all three models together (3-model consensus). In general, researchers acknowledged an increase in overall predictive performance, if consensus of predictions among a battery of models was applied [69]. Following the protocol of Marzo et al. [69] we made various combinations of consensus of model predictions by combining predictions from NN-C, NN-D and Q-D models. The 3-model consensus A was the strictest and considered only predictions with all three models (NN-C + NN-D + Q-D) in agreement. In 3-model consensus ‘A + B’ the predictions with maximal one disagreement among the NN-C, NN-D and Q-D models were considered. In 2-model consensus ‘NN-D + Q-D’ the predictions where both models were in agreement were considered. In the same way the rest two combinations of a 2-model consensus (NN-C + NN-D and NN-C + Q-D) were also performed, but have lowest performance evaluation than ‘NN-D + Q-D’. The results of performance evaluation of consensus modeling are represented in Table 1 and Table 2, and the detailed predictions in Table S1 (Supplementary Material). When compared with the single models, the accuracy performance of consensus of predictions was increased, but consequently the prediction rate was decreased. The highest overall accuracy (0.99) was determined in 2-model consensus ‘NN-D + Q-D’ and 3-model consensus A, yet both consensus significantly differ in prediction rate ability, 81% and 62%, respectively. Therefore, the best balance among accuracy and prediction rate was achieved with 3-model consensus ‘A + B’ (ACC = 0.92, PR = 100%). This 3-model consensus was shown as robust and reliable, and in evaluation parameters outperformed all single models. In this regard, the high impact of integration of predictions of three models was conformed and in concordance with expectations the overall predictive performance was increased.

#### 2.1.2. Application of Classification Models on Phenolic Dataset

**Figure 5:**
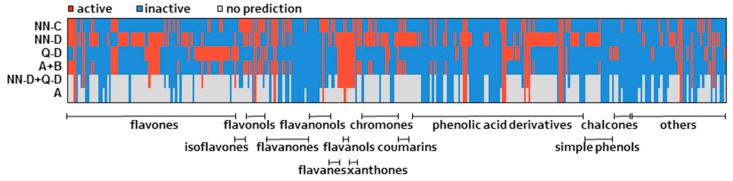
Representation of classification of 300 compounds with three separate classification models (NN-C, NN-D, Q-D) and three consensus models (A + B, NN-D + Q-D, A) on graphical map.

Developed QSAR models enabled efficient application in screening processes regarding bioavailability of dietary/pharmaceutically interesting compounds. With single classification models and 2-/3-model consensus of predictions we estimated the BTL transport potential for compounds, which were not experimentally tested for BTL transport activity (phenolic dataset, *n* = 300). Results of predictions are represented in Figure 5 and Table S2 (Supplementary Material). Consensus ‘A + B’ and single models N-C and Q-D performed with a 100% prediction rate with most of the compounds within AD (A + B = 300, NN-C = 283, Q-D = 278). On the other hand, the model NN-D resulted with a lower number of compounds in AD (NN-D = 208). As expected, lower prediction rate was evaluated for other consensus of predictions (NN-D + Q-D = 50%, A = 36%), due to strictest conditions. Generally, the integration of multiple models increased the overall reliability of predictions in all consensus combinations, also increased the prediction rate for phenolic compounds in consensus ‘A + B’, but decreased in other consensus (NN-D + Q-D, A). 

Using in silico models one is challenged with the paradigm of selecting single model or very strict consensus (e.g., A) with high accuracy and narrow AD, or on the price of broadening of AD decide for wider consensus (e.g., A + B). In this regard, the number of active compounds predictions varied from 15 in consensus A to 65 in consensus ‘A + B’ (Table S2, Supplementary Material). Among single models the highest number of active compounds was predicted with the model NN-D (138), which was significantly higher than in other models (NN-C = 75, Q-D = 72). However, none of single models or consensus of predictions did not recognize groups of phenols that are more likely to interact with BTL (Figure 5). For sure the most promising active compounds are those 15 that were predicted in all models: luteolin (ID4), kaempferol (ID86), eriodictyol (ID95), pinobanksin (ID117), cianidanol (ID127), leucodelphinidin (ID131), ellagic acid (ID181), rosmarinic acid (ID182), gallic acid (ID199), methyl gallate (ID200), 3-methoxy-4-hydroxybenzoic acid (ID209), 3-methoxy-4-hydroxyhippuric acid (ID211), decanyl caffeate (ID225), oleuropein (ID226), PACD3 (ID280) (see Table S2, column A, Supplementary Material). Since in vivo/in vitro experimental data on membrane transport ability for majority of the above mentioned compounds are not available either for BTL neither for other transporters from the list in Table 3 (except for four compounds and few transporters, see results in Section 2.3), the in silico predictions are currently the only relevant estimations of their membrane transport activities. Interestingly, based on our results BTL might be also involved in the gastric absorption of the flavone luteolin, as its gastric uptake was observed in vivo [70]. Indeed, this is in support of hypothesis of Passamonti et al. [35] that at the gastric level the BTL-mediated mechanism could play an important role in active transport of polyphenols. The isoflavone daizdein, on the other hand, was classified as inactive with our consensus of QSAR models and was also experimentally identified as not being a BTL substrate [35]. However, study of Piskula et al. [71] demonstrated that daizdein can be absorbed from the rat stomach, thus in its gastric uptake probably another specific OAT transporter, a monocarboxylate transporter, is involved [35].

### 2.2. BTL Regression Models

The activity predictions for the BTL inhibition constants pKi [mmol/L] were performed with the best in-house predictive CP-ANN models (M1–M4) [59]. The neural networks were selected regarding results from the previous studies [59]. Firstly, we analyzed, if the regression models were accurately developed and validated regarding the need of evenly frequency distribution of compounds in all ranges of BTL biological activity in the training and validation datasets. This condition was fully satisfied and a compounds frequency distribution in five intervals of BTL activity is shown in Figure S1 (Supplementary Material). For further detailed evaluation of models a five quality parameters of validation were employed (R^2^, RMSE, Q^2^_F3_, CCC, r_m_^2^), and their performance is represented in Table 4. Statistical differences among models are low and in general all validation parameters characterize model M1 as the best one. The validation parameter r_m_^2^ was specially reported to help in identifying the best models among a set of comparable models [72], but in this case all parameters were equally sensitive. Since GA approach was used in the development and optimization of models, consequently a large availability of different models predicting the same endpoint was produced. Thus, to avoid having cluster of similar models we tested the diversity of the top scoring models by measuring of Hamming distance between them. The results are shown in Table S5 (Supplementary Material). The distances among models are in range of 7–17. This indicated that numbers of variables not shared by the models are high. Accordingly, our set of top models was shown to be diverse and consensus analyses were justified. An average response and weighted average response were used as proposed by Todeschini et al. [65]. The consensus gave better statistical fits of predictions in regard to the single models.

#### Application of Regression Models on Phenolic Dataset

Predictions were made for 205 compounds. These were compounds that were classified as active with at least one model (Table S7, Supplementary Material). In order to produce more reliable estimates of our endpoint the consensus modeling strategy was also applied. Most of models were well applicable for phenols, since majority of compounds were in AD of models (>75%); exception was model M3 with only 50% of compounds in AD. Moreover, the predictions were highly harmonized, since varied for less than 10% of standard deviation, if compared among all models (M1-M4). Up to our knowledge only one study exist on extrapolation of BTL models to make predictions of new compounds, which were not included in model development or validation [59]. Comparison with that study revealed that BTL models have wider capacity for prediction of phenolic compounds than other antioxidants and antiprions (Table S6, Supplementary Material). 

### 2.3. Metrabase and Chembench Analyses

**Figure 6:**
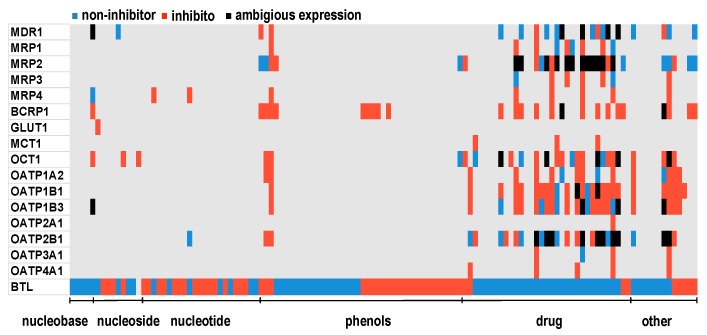
Activity profile for 120 compounds including BTL experimental data [53,54] and data available for 16 transporters in Metrabase [46]. *Grey*—no data available in database.

**Figure 7:**
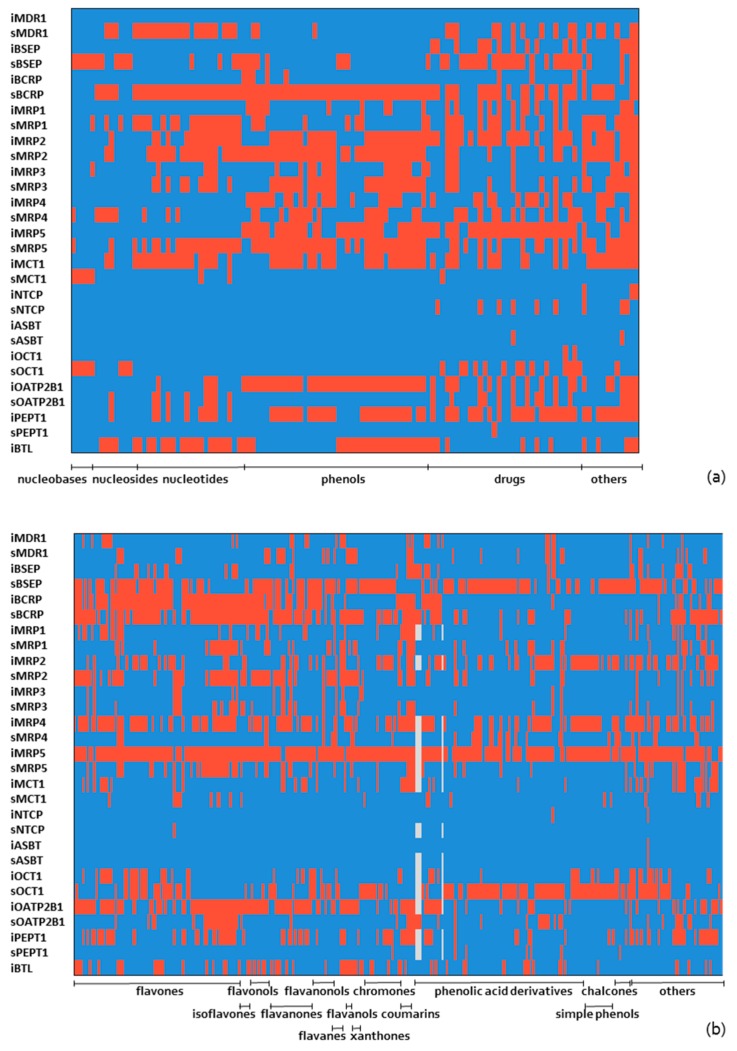
Activity profile of computational predictions for 14 transporters from ChemBench and for our BTL model, *red—*active, *blue*—inactive, *grey*—no prediction; (**a**) BTL dataset of 120 compounds, (**b**) phenols dataset of 300 compounds.

Based on data and tools available on Metrabase and Chembench platforms we made the comprehensive virtual profiling of membrane transport interactions for compounds of both datasets. Herein, we performed the unique transporter profile of studied compounds, which is based on the results from new BTL models and hits/predictions from over 20 membrane transporters of Metrabase and Chembench (Figure 6 and Figure 7). 

We compared the transport activity data among BTL and 16 transporters that are publicly available in Metrabase (cheminformatics and bioinformatics database) [46]. Analysis of hits of BTL dataset (compounds from Table S1, Supplementary material) in Metrabase database is represented on Figure 6. It was found out that almost no experimental data exist for the phenolic compounds (ID 37–84), nucleobases, nucleosides and nucleotides (ID 1–36). The only exception was few flavonoids (ID 65–70). On the other hand, drugs and endogenous substances (ID 85–120), which are of interest for pharmaceutical industry, were better studied and experimentally analyzed, therefore more hits were found. 

Based on these results we can conclude that for phenolic compounds, nucleobases, nucleosides and nucleotides the only available information regarding experimental transport activity of the membrane transporters is those of BTL provided from research group of prof. Passamonti [53,54], which are not included in Metrabase. Further on, we made analyses in Metrabase for compounds of consensus A, which were predictive as active (15 compounds, see Section 2.1.2). We found out that only for luteolin (ID4), kampferol (ID86), cianidanol (ID127) and eriodictyol (ID95) a few experimental studies exist in this database; luteolin (MDR1–inhibitor, BCRP1–inhibitor, MRP2–non-inhibitor), kaempferol (BCRP1–inhibitor, MRP2–non-inhibitor, OCT1–inhibitor, OATP1A2–inhibitor), cianidanol (MRP2–non-inhibitor or substrate, OCT1–non-inhibitor, OATP2B1–inhibitor or non-inhibitor) eriodictyol (MRP2–non-inhibitor). Besides this four compounds, which were captured the significant attention of the scientific audience for their potential anti-oxidative, anti-tumor, and anti-inflammatory effects, also other phenolic compound of natural origin like oleuropein (ID226) and leucodelphinidin (ID131) are of interest due to their anti-neurodegenerative and hypolipidaemic effects [73,74]. Thus, the predictions about transport ability by BTL and 14 others importers and exporters that are represented in Tables S2 and S9 (Supplementary Material) are of high interest for clarification about their active transport across membrane. Further on, we made predictions for 420 compounds (BTL and phenolic datasets) by using QSAR models of 14 transporters available in Human intestinal transporter database at Chembench platform [44]. The results of predictions are represented in Figure 7, Tables S8 and S9 (Supplementary Material). In general, the models have been found to have good prediction ability and coverage for phenolic compounds, since only few compounds were out of AD or unable to predict (Table S9, Supplementary Material). Yet, almost all models had poor prediction ability for nucleobases, nucleosides and nucleotides (ID 1–36, Table S8, Supplementary Material). Poor prediction ability was observed also for some flavonoids in models of MRP4, MRP5, MCT1, NTCP, ASBT and OCT1 (anthocyanidins (ID 71–84) and flavonols (ID 52–64)). 

## 3. Discussion

In spite a lack of adequate experimental validation for our in silico predictions, some information could be gained by comparing structurally similar compounds. Among the fifteen the most promising active compounds, which were selected with the BTL QSAR models, luteolin, kaempferol, eriodictyol and pinobanksin were found to share the highest structural similarity with quercetin. Quercetin, malvidin 3-glucoside, bilirubin and bromosulfophthalein are up to date the only compounds identified experimentally as BTL substrates [55,75]. Hopefully, the results published in this study would be inspiration for in vitro experts to test the most promising predicted active compounds with the BTL transport activity assay. The compounds of natural origin with high health benefit should be selected first, like oleuropein, luteolin, eriodictyol, cianidanol, and leucodelphinidin.

The clinic reports highlighted the impact of knowledge about how various therapeutics or dietary supplements interacts with membrane transporters [76]. Herein, for the first time were represented joint analyses of transport activities of BTL and several others membrane transporters. Results not solely present a new insight in interactions between phenols and several membrane transporters, but can help also in assessment of their potential absorption and disposition properties, yet gain also valuable information for understanding possible interference of phenols with the transport activity of drugs. Since ingestion of extra dietary phenolic supplements as support to chemotherapy was proposed as promising therapeutic approach [7,10], but such diet could consequently influence drugs absorption and disposition [58]. For proper drug application this is for sure of high importance. Moreover, results of the systematic analysis performed in this study are also important for elucidation of functional role of BTL, as up to our knowledge only limited data exist. Up to date, only one synopsis of reported interactions with BTL and other membrane transporters one a few compounds (>50) is available [58]. Since in general not much is known about interactions between phenols and transporters, the results from this study are of interest [77]. Moreover, we managed to confirm our expectations, that newly developed BTL QSAR models have good predictive ability and coverage for phenolic compounds. In future it would be of interest to make a similar study on the pharmaceutically interested nucleobase derivatives [78,79,80], for which it is assumed that BTL could have important role on their transport [54]. 

More than half a century has passed since an implementation of Hansch-Fujita approach in the formulation of QSAR, which provided a new perspective for chemical-biological interactions and a number of successful applications are known especially in drug discovery [77]. Nowadays, we have abundance of QSAR models for predictions of numerous endpoints in various fields of science. In this study we tried to collect and compare the QSAR models of membrane transporters to make a comprehensive transporter profiles for purposes of virtual screening of compounds for their bioavailabilty. Now point of future investigation should be to switch in new level beyond individual approaches and integrate various QSAR analyses with ontology and omics data [77,81,82,83]. Such simultaneously predicting with joint data analyses are shown to increase the accuracy of models by exploiting their common representation and identifying common features between individual properties, which are frequently strongly correlated with one another [81,83]. For sure not in so far future a chemical characterization will be even more empowered with an artificial intelligence assisted software, which would make decisions based on integration of numerous data from in silico, in vitro, in vivo and in situ analyses [84]. Thus, hopefully models from this study will serve like small piece of a large puzzle in the complex biochemical system analysis.

## 4. Materials and Methods 

Quantitative structure-activity relationships (QSARs) are widely used in silico methods to predict biological activity for untested chemicals (Figure 1). This is proven, time and cost-effective approach, which is in most cases sufficient alternative method to animal testing [44]. For building and validation of models the appropriate statistical algorithms and the data matrix which includes numerical values of chemical structures and empirical values of biological activity are needed. 

### 4.1. Dataset

The dataset for modeling included 120 aromatic organic compounds (43 phenols and 77 various aromatic compounds like nucleobase derivatives, drugs, dyes, etc.) [59]. In general chemical structures of all compounds were optimized and numerically coded with molecular descriptors (MD). The MD calculation and selection of influential MD is described in the Section 4.2.1. The corresponding empirical data of BTL transport activity assay were collected from experiments of Župerl et al. [54] and Karawajczk et al. [53]. Inhibition constants (*K_i_*) were determined based on Michaelis-Menten constants, which were obtained from spectrophotometrical evaluation of BTL transport activity on selected compounds in medium of sulfobromophtalein (BSP) and rat liver plasma membrane vesicles. According to threshold criteria proposed by Župerl et al. [54] the dataset composed of 50 active compounds (inhibitors, *pK_i_* > 1.3) and 70 inactive compounds (non-inhibitors, *pK_i_* ≤ 1.3). The compounds are listed in Table S1 (Supplementary Material). The test dataset was compiled from literature. It encompasses natural occurring and synthetic phenolic compounds having various potential health benefits. Altogether 300 compounds were collected from over 30 publications [7,11,12,13,14,15,16,17,18,19,20,21,22,23,24,25,26,27,28,29,30,85,86,87,88,89,90,91,92,93], see Table S2 (Supplementary Material). Compounds were arranged according to their structural similarity into 12 groups: flavones/isoflavones, flavonols, flavanones/ isoflavanones, flavanonols, flavanols, xanthones, chromones, coumarins, chalcones, phenolic acid derivatives, simple phenols and other compounds. Experimental evaluation of potential role of selected phenolic compounds in preventing various chronic diseases are explained in detail in the references listed in Table S2 (Supplementary Material).

### 4.2. Computational Analysis

#### 4.2.1. Molecular Descriptors

Molecular structures were represented with a set of molecular descriptors (MDs). First, the MOPAC software [94] was used for optimization of the 2D chemical structures into 3D conformations considering the minimal energy criterion for the optimal conformation of the compound in vacuum. Then numerical coding of 3D conformations was performed with MDs, which were calculated with the CODESSA program [95]. With the applied software more than 400 MDs for each compound were calculated. Second, the numerical encoding of 2D conformations was performed with Dragon 7.0 software (Kode srl., Pisa, Italy) [96], which calculated over 3000 MDs for each molecule. The dataset with Codessa and Dragon descriptors were further on treated separately. So, obtained two datasets were normalized to zero mean and unit standard deviation for each descriptor. Afterwards, the reduction of numerous descriptors was performed by omitting the MDs with zero variance or high intercorrelation (>0.9). The set of descriptors was then reduced according to the similarity criterion in the Kohonen map by following the protocol of Martinčič et al. [59], which resulted in the final data pool of 66 and 87 MDs for Codessa and Dragon dataset, respectively.

#### 4.2.2. Generation of Models

The inputs for model building were various *m*-dimensional vectors representing the chemical structure, *m* being the number of MDs selected (independent variables), and the targets (properties, i.e. dependent variables) corresponding to p*K_i_* of BTL transport activity. We used CPANNatNIC software for the splitting of data for modeling into training and validation set [59,92]. For model fitting the linear and nonlinear regression approaches were used. The multiple linear regression models were developed with Qsarins software (DiSTA, Varese, Italy, www.qsar.it) [68]. The supervised learning models were developed by using counter-propagation artificial neural network (CP-ANN) algorithm and in-house software [97,98]. For classification and prediction of unknown compounds two QSAR modeling strategies were adopted. Firstly, the classification models were developed to separate chemicals into active/non-active classes, and then optimized regression models were used for the active chemicals to predict the values of the inhibition constants [59]. All 120 compounds were considered for the construction and optimization of the classification model, while the predictive models are based on active compounds only. For optimization we applied cross-validation (CV) and genetic algorithm (GA) [65,98] in order to select the influential descriptors and to improve the predictive ability and robustness of the models. In models selection the applicability domain (AD) [67] was applied to evaluate the model predictions within the established chemical space limits. 

#### 4.2.3. Statistical Evaluation of Models 

The criteria for selection of the best classification models, so called quality indicators, are listed in Table 5 [99,100]. 

Models with the highest quality indicators of the validation set and wider applicability (larger AD) were further analyzed. Two approaches were used for AD evaluation, the cumulative distributions of Euclidian distances to central neurons (MEDS) for CP-ANN models [67] and the leverage values for MLR models [68]. Several models were built by each of the selected modelling method. Further on, the consensus of model predictions was performed by combining predictions of three or two QSAR models. The consensus was performed following the protocol of Marzo et al. [69], which including arranging of compounds in two types: A—complete agreement among predictions, B-maximum one disagreement among the predictions.

The criteria for selection of the best regression models were several validation parameters (R^2^, RMSE, CCC, Q^2^_F3_, r_m_^2^) [64,70]. The consensus approach was also applied for the best four regression models (M1-M4) by using the weighted average response. The calculation was performed following the Equation (1); M—number of models, *y_k_*—response estimated by the *k*-th model, *h_k_*—leverage [65]:

(1) $$\bar{y_{w}}=\;\frac{\sum_{k=1}^{M}\frac{y_{k}}{h_{k}}}{\sum_{k=1}^{M}\frac{1}{h_{k}}}$$

In order to evaluate diversity of models the Hamming distances *d_h_* between models were accounted based on the two-way table [65] (Equation (2)). The table represent the relationship between two binary vectors, each vector represent one model; *b*—the number of cases that for same position there is 1 in vector I and 0 vector II, *c*—the number of cases that for same position there is 0 in vector I and 1 vector II Values in Table S5 (Supplementary Material) represent the number of MD not shared among models:

*d_h_* = *b* + *c* (2)

### 4.3. Metrabase and Chembench Analyses 

The public available cheminformatics and bioinformatics database for small molecule transporter data analysis (Metrabase) [46,101] was screened for transport activity profile of 120 compounds (BTL dataset). The synopsis of transport activity among BTL and 16 transporters from Metrabase was performed. The selected 16 transporters are listed in Table 3. Further on, in Human intestinal transporter database, available on Chembench platform, the QSAR models of 14 transporters were used to make predictions for 420 compounds (BTL and phenolic datasets) [44,102]. The transporters are listed in Table 3 and detailed list of QSAR models properties is summarized in Table S3 (Supplementary Material). The comparison of transport activity among BTL and 14 transporters from Chembench was performed for both datasets.

## 5. Conclusions

New classifiers for BTL transport activity were developed. Based on in silico approach for estimation of BTL transport activity the classification for dietary/pharmaceutically interesting compounds (natural dietary phenols and synthetic phenol-like drugs) were performed. Using QSAR classification models (NN-C, NN-D, Q-D) we estimated the BTL transport activity of 300 compounds, which have not been previously experimentally evaluated. Moreover, the consensus among battery of models was applied, since the detailed evaluation of models shown better performance than individual models. With various predictive CP-ANN models (M1-M4) then the transport activity prediction (inhibition constant pKi [mmol/L]) for the compounds classified as active was assessed. The reliability of all predictions was evaluated with the applicability domain (AD) of each individual classification and regression model. In general, BTL is known as a potential delivery system for wide variety of the vitally important endogenous substrates and drugs, yet with the models represented in this study a good ability for predicting phenolic compounds was shown too. The 205 compounds from the dataset of 300 phenols have a potential to interact with BTL.

The central part of this study was the synopsis of unique transporter profiles of BTL in comparison with 20 other membrane transporters from Metrabase and Chembench. Results present a new insight in interactions between phenols and several membrane transporters, which can help not solely in assessment of their potential absorption and disposition properties but also gain insight in possible interference of phenols with the transport activity of drugs, what can be valuable information in drug application.

## Figures and Tables

**Table 1:** Statistic parameters of the best three single models and consensus classification models.

	Single Models			2-Model Consensus			3-Model Consensus		
	NN-C	NN-D	Q-D	NN-C + NN-D	NN-C + Q-D	NN-C + Q-D	A	B	A + B
**TP**	44	44	46	33	33	41	29	19	48
**TN**	61	65	58	47	49	55	45	17	62
**FP**	9	5	12	6	2	1	1	7	8
**FN**	6	6	4	0	2	0	0	2	2
**∑**	120	120	120	86	86	97	75	45	120
**PR**	1 (0.98)	1 (0.92)	1 (0.97)	0.72	0.72	0.81	0.62	0.37	1
**SE**	0.88	0.88	0.92	1.00	0.94	1.00	1.00	0.90	0.96
**SP**	0.87	0.93	0.83	0.89	0.96	0.98	0.98	0.71	0.89
**ACC**	0.88	0.91	0.87	0.93	0.95	0.99	0.99	0.80	0.92
**NPV**	0.91	0.92	0.94	1.00	0.96	1.00	1.00	0.89	0.97
**PPV**	0.83	0.90	0.79	0.85	0.94	0.98	0.97	0.73	0.86
**MCC**	0.75	0.81	0.74	0.87	0.90	0.98	0.97	0.62	0.84

A—complete agreement among predictions, B—maximal one disagreement among predictions.

**Table 2:** Summary of classification models results.

Model ID.	Type	No. of Descriptors	ACC_TR_	ACC_V_	Predict Ratio [%]		No. of Active Compound Predictions	
					*BTL Dataset*	*Phenols*	*BTL Dataset*	*Phenols*
NN-C	CP-ANN	66	0.93	0.74	100 (97)	100 (94)	54 (51)	75 (74)
NN-D	CP-ANN	18	0.92	0.87	100 (92)	100 (69)	49 (46)	138 (100)
Q-D	MLR	11	0.88	0.77	100 (97)	100 (93)	58 (55)	72 (68)
A + B	3 cons	/	0.93	0.87	100 (99)	100 (100)	56 (55)	65 (65)
A	3 cons	/	1.00	0.92	62 (62)	36 (36)	30 (30)	15 (15)
NN-D + Q-D	2 cons	/	1.00	0.96	81 (81)	50 (50)	42 (42)	31 (31)

( ) values for compounds in AD; 3 cons—3-model consensus; 2 cons—2-model consensus.

**Table 3:** List of transporters from Metrabase and ChemBench platforms.

Protein Symbol	Gene Symbol	Name	Tissue Expression Data
**MDR1 ^M,C^**	ABCB1	ATP-binding cassette sub-family B member 1, P-glycoprotein	intestine, kidney, liver, brain
**BSEP ^C^**	ABCB11	ATP binding cassette subfamily B member 11	liver
**MRP1 ^M,C^**	ABCC1	ATP-binding cassette, sub-family C member 1	intestine, kidney, liver, brain
**MRP2 ^M,C^**	ABCC2	ATP-binding cassette sub-family C member 2	stomach, intestine, kidney, liver, brain
**MRP3 ^M,C^**	ABCC3	ATP-binding cassette sub-family C member 3	stomach, intestine, kidney, liver, brain
**MRP4 ^M,C^**	ABCC4	ATP-binding cassette, sub-family C member 4	stomach, intestine, kidney, liver, brain
**MRP5 ^C^**	ABCC5	ATP Binding Cassette Subfamily C member 5	kidney, brain
**BCRP1 ^M,C^**	ABCG2	ATP-binding cassette sub-family G member 2	stomach, intestine, kidney, liver, brain
**OATP1A2 ^M^**	SLCO1A2	solute carrier organic anion transporter family member 1A2	kidney, liver, brain
**OATP2A1 ^M^**	SLCO2A1	solute carrier organic anion transporter family member 2A1	intestine, kidney, liver, brain
**OATP1B1 ^M^**	SLCO1B1	solute carrier organic anion transporter family member 1B1	liver
**OATP1B3 ^M^**	SLCO1B3	solute carrier organic anion transporter family member 1B3	liver
**OATP2B1 ^M,C^**	SLCO2B1	solute carrier organic anion transporter family member 2B1	intestine, kidney, liver, brain
**OATP3A1 ^M^**	SLCO3A1	solute carrier organic anion transporter family member 3A1	intestine, kidney, liver, brain
**OATP4A1 ^M^**	SLCO4A1	solute carrier organic anion transporter family member 4A1	stomach, intestine, kidney, liver, brain
**GLUT1 ^M^**	SLC2A1	solute carrier family 2 (facilitated glucose transporter) member 1	intestine, kidney, liver, brain
**NTCP ^C^**	SLC10A1	solute carrier family 10 member 1	liver
**ASBT ^C^**	SLC10A2	solute carrier family 10 Member 2	intestine
**PEPT1 ^C^**	SLC15A1	solute carrier family 15 member 1	intestine
**MCT1 ^M,C^**	SLC16A1	solute carrier family 16 member 1, monocarboxylic acid transporter 1	stomach, intestine, kidney, liver, brain
**OCT1 ^M,C^**	SLC22A1	solute carrier family 22 (organic cation transporter) member 1	intestine, kidney, liver
BTL *	/	bilitranslocase, bilirubin membrane transporter	stomach, intestine, kidney, liver

* new, not available in Metrabase and ChemBench, M—available in Metrabase, C—available in ChemBench.

**Table 4:** Statistic parameters of the best four regression models.

Model	R^2^_tr_	RMSE_iv_	R^2^_iv_	Q^2^_F3iv_	CCC_iv_	R^2^_ev_	Q^2^_F3ev_	CCC_ev_	RMSE_all_	CCC_all_	r_m_^2^_(all)_
**M1**	0.996	0.266	0.949	0.957	0.970	0.872	0.927	0.939	0.198	0.987	0.821
**M2**	0.999	0.280	0.943	0.953	0.968	0.842	0.909	0.927	0.206	0.986	0.812
**M3**	0.998	0.289	0.939	0.950	0.964	0.839	0.908	0.934	0.213	0.985	0.806
**M4**	0.995	0.295	0.937	0.948	0.964	0.760	0.863	0.899	0.243	0.980	0.776

**Table 5:** Quality indicators for evaluation of different classification models.

Acronym	Quality Indicator	Formula
**TP**	true positive	
**TN**	true negative	
**FP**	false positive	
**FN**	false negative	
**PR**	predict ratio	predicted compounds/total compounds
**SE, TPR**	sensitivity, true positive rate	TP/(TP + FN)
**SP, TNR**	specificity, true negative rate	TN/(TN + FP)
**ACC**	accuracy	(SP + SE)/2
**NPV**	negative predictive value	TN/(TN + FN)
**PPV**	positive predictive value, precision	TP/(TP + FP)
**MCC**	Matthews correlation coefficient	((TP*TN)-(FP*FN))/√[(TP + FP)(TP + FN)(TN + FP)(TN + FN)]

## Supplementary Materials

The list of ChemBench models and detailed list of BTL and phenolic datasets with extended results of data analyses (Tables S1–S9) are available as supplementary material online.
