# Supplementary material for: An In Silico Approach for Assessment of the Membrane Transporter Activities of Phenols: A Case Study Based on Computational Models of Transport Activity for the Transporter Bilitranslocase
Source: Molecules. 2019 Feb 27;24(5):837. doi: 10.3390/molecules24050837 (PMC6429229; doi:10.3390/molecules24050837)
Supplement: Supplementary file 1 [file molecules-24-00837-s001.pdf]

SUPPLEMETARY MATERIAL

In Silico Approach for Assessment of Membrane Transporters Activities of Phenols: Case Study Based on Computational Models of Transport Activity for Transporter Bilitranslocase

Katja Venko and Marjana Novič

Table S1: List of 120 chemicals with BTL activity experimental values (target) and the classification predictions of separate models (NN-C, NN-D, Q-D) and consensus of models (A+B, NN-D+Q-C, A).

ID	Name	Target (exp.)	NN-D	NN-D	Q-C	AB prediction	A+B	NN-D + Q-D	A
**1**	Adenine	**0**	0	0	0	A	0	0	0
**2**	Adenosine	**0**	0	0	1	B	0	/	/
**3**	Adenosine 3′-monophoshate	**1**	0	1	1	B	1	1	/
**4**	Adenosine 5′-monophoshate	**1**	1	1	1	A	1	1	1
**5**	Adenosine 3′, 5′-cyclic monophosphate	**0**	0	0	0	A	0	0	0
**6**	Adenosine 5′-diphosphate	**1**	0	1	1	B	1	1	/
**7**	Adenosine 5′-triphosphate	**1**	1	0	1	B	1	/	/
**8**	Adenosine-5′-diphosphoglucose	**0**	0	0	0	A	0	0	0
**9**	Adenosine 5'-(α,β-methylene) diphosphate	**1**	1	1	1	A	1	1	1
**10**	Adenine 9-β-D-arabinofuranoside	**1**	1	0	1	B	1	/	/
**11**	Adenosine 3′-phosphate 5′-phosphosulfate	**1**	0	1	1	B	1	1	/
**12**	S-(5′-Adenosyl)-L-homocysteine	**1**	1	1	0	B	1	/	/
**13**	S-(5′-Adenosyl)-L-methionine chloride	**1**	1	1	0	B	1	/	/
**14**	Guanosine	**0**	1	0	1	B	1	/	/
**15**	Guanosine 5′-monophosphate	**1**	1	1	1	A	1	1	1
**16**	Guanosine 3′. 5′-cyclic monophosphate	**0**	0	0	0	A	0	0	0
**17**	Guanosine 5′-diphosphate	**1**	1	1	1	A	1	1	1
**18**	Guanosine 5′-triphosphate	**1**	1	1	1	A	1	1	1
**19**	Uracil	**0**	0	0	0	A	0	0	0
**20**	Uridine	**1**	0	0	1	B	0	/	/
**21**	Uridine 5′-monophosphate	**1**	1	0	1	B	1	/	/
**22**	Uridine 5′-diphosphate	**1**	1	1	1	A	1	1	1
**23**	Uridine 5′-triphosphate	**1**	1	1	1	A	1	1	1
**24**	Uridine 5'-diphosphoglucose	**0**	0	1x	0	B	0	/	/
**25**	Uridine 5'-diphosphogalactose	**1**	1	1x	0	B	1	/	/
**26**	Uridine 5′-diphosphoglucuronic acid	**0**	1	1	0	B	1	/	/
**27**	Thymine	**0**	1	0	0	B	0	0	/
**28**	Thymidine	**0**	0	0	0	A	0	0	0
**29**	Thymidine 5′-monophosphate	**1**	1	1	1	A	1	1	1
**30**	Thymidine 5′-diphosphate	**1**	1	1	1	A	1	1	1
**31**	Thymidine 5′-triphosphate	**1**	1	1	1	A	1	1	1
**32**	Cytosine	**0**	0	0	0	A	0	0	0
**33**	Cytidine	**0**	0	0	1	B	0	/	/
**34**	Cytidine 2′-monophosphate	**0**	1	0	0	B	0	0	/
**35**	Cytidine 5′-monophosphate	**0**	1	0	1	B	1	/	/
**36**	Uric acid	**1**	0	0	1	B	0	/	/
**37**	Ouabain	**0**	0	0	0	A	0	0	0
**38**	Aucubin	**0**	0	0	0	A	0	0	/
**39**	Loganin	**0**	1	0	1	B	1	/	/
**40**	Verbenalin	**0**	0	0	1	B	0	/	/
**41**	Isovitexin	**0**	0	0	0	A	0	0	0
**42**	Vitexin-2'-O-rhamnoside	**0**	0	0	0	A	0	0	0
**43**	Cibacron Blue F3G-A	**1**	1	1	1	A	1	1	1
**44**	Digoxin	**0**	0	0	0	A	0	0	0
**45**	Taurocholate	**0**	0	0	0	A	0	0	0
**46**	Pelargonidin	**1**	1	1	1	A	1	1	1
**47**	Cyanidin	**1**	0	1	1	B	1	1	/
**48**	Delphinidin	**1**	1	1	1	A	1	1	1
**49**	Peonidin	**1**	1	1	1	A	1	1	1

**50**	Petunidin	**1**	1	1	1	A	1	1	1
**51**	Malvidin	**1**	0	1	1	B	1	1	/
**52**	Pelargonidin 3-O-β-D-glucopyranoside	**1**	0	1	1	B	1	1	/
**53**	Cyanidin 3-O-β-D-glucopyranoside	**1**	1	1	1	A	1	1	1
**54**	Delphinidin 3-O-β-D-glucopyranoside	**1**	1	1	1	A	1	1	1
**55**	Peonidin 3-O-β-D-glucopyranoside	**1**	1	1	1	A	1	1	1
**56**	Petunidin 3-O-β-D-glucopyranoside	**1**	1	1	1	A	1	1	1
**57**	Malvidin 3-O-β-D-glucopyranoside	**1**	0	1	1	B	1	1	/
**58**	Pelargonidin 3,5-di-O-β-D-glucopyranoside	**1**	0	1	1	B	1	1	/
**59**	Cyanidin 3,5-di-O-β-D-glucopyranoside	**1**	1	1	1	A	1	1	1
**60**	Peonidin 3,5-di-O-β-D-glucopyranoside	**1**	0	1	1	B	1	1	/
**61**	Malvidin 3,5-di-O-β-D-glucopyranoside	**1**	1	1	1	A	1	1	1
**62**	Cyanidin 3-O-α-L-arabinopyranoside	**1**	1	1	1	A	1	1	1
**63**	Cyanidin 3-O-β-D-galactopyranoside	**1**	1	1	1	A	1	1	1
**64**	Malvidin 3-O-β-D-glucopyranoside	**1**	1	1	1	A	1	1	1
**65**	Galangin	**1**	1	1	0	B	1	/	/
**66**	Kaempferol	**1**	0	1	1	B	1	1	/
**67**	Quercetin	**1**	1	0	1	B	1	/	/
**68**	Myricetin	**0**	0	0	1	B	0	/	/
**69**	Syringetin	**0**	0	0	0	A	0	0	0
**70**	Rhamnetin	**0**	0	0	0	A	0	0	0
**71**	Quercetin 4’-glucopyranoside	**0**	1	0	0	B	0	0	/
**72**	Quercetin 3,4’-diglucopyranoside	**0**	0	0	0	A	0	0	0
**73**	Quercetin 3-glucopyranoside	**0**	0	0	0	A	0	0	0
**74**	Quercetin 3-xyloside	**0**	0	0	0	A	0	0	0
**75**	Quercetin 3-rhamnoside	**0**	0	0	0	A	0	0	0
**76**	Quercetin 3-galactoside	**0**	1	0	0	B	0	0	/
**77**	Quercetin 3-O-glucopyranosyl-6'-acetate	**0**	0	0	0	A	0	0	0
**78**	Quercetin 3-O-sulfate	**0**	0x	0	0	A	0	0	0
**79**	Isorhamnetin 3-glucoside	**0**	1	0	0	B	0	0	/
**80**	Isorhamnetin 3-O-rutinoside	**0**	0	0	0	A	0	0	0
**81**	Kaempferol 3-O-glucoside	**0**	0	0	0	A	0	0	0
**82**	Kaempferol 3-O-rutinoside	**0**	1	0	0	B	0	0	0
**83**	Syringetin 3-galactoside	**0**	0	0	0	A	0	0	0
**84**	Syringetin 3-glucoside	**0**	0	0	0	A	0	0	0
**85**	Nicotinic Acid	**0**	0	0	0	A	0	0	0
**86**	[D-Ala2]- Deltorphin II	**0**	1x	0x	1x	B	1x	/	/
**87**	[D-Pen2.5]-Enkephalin (DPDPE)	**0**	1	0	0	B	0	0	/
**88**	17β-Estradiol 3-glucuronide	**0**	0	0	0	A	0	0	0
**89**	5-Fluorouracil	**0**	0	0	0	A	0	0	0
**90**	Acetazolamide	**0**	0x	0x	0	A	0	0	0
**91**	Acycloguanosine (Acyclovir)	**0**	1	0	0	B	0	0	/
**92**	Etacrynic Acid	**0**	0	0x	0	A	0	0	0
**93**	Mefenamic Acid	**0**	0	0	0	A	0	0	0
**94**	Dehydroisoandrosterone 3-sulfate	**0**	0	0	0	A	0	0	0
**95**	Diclofenac	**0**	0	0	0	A	0	0	0
**96**	Dichlorofluorescein (DCFH)	**0**	0	0	0	A	0	0	0
**97**	Enalapril maleate	**0**	0	0	0x	A	0	0	0
**98**	Estrone 3-sulfate	**0**	0	0	0	A	0	0	0
**99**	Phenacitin	**0**	0	0	0	A	0	0	0
**100**	Furosemide	**0**	0	0	0	A	0	0	0
**101**	Glycocholate	**0**	1	0x	0	B	0	0	/
**102**	Ibuprofen	**0**	0	0x	0	A	0	0	0
**103**	Hydrochlorothiazide	**1**	1	1	1	A	1	1	1
**104**	Hydrocortisone	**0**	1	0	1	B	1	/	/
**105**	Indomethacin	**0**	0	1	0	B	0	/	/
**106**	Ketoprofen	**0**	1	0	0	B	0	0	/
**107**	L-Thyroxine (T4)	**0**	0	1	1x	B	1	1	/
**108**	Methotrexate	**0**	0	0x	1	B	0	/	/
**109**	Naproxene	**0**	0	0	0	A	0	0	0
**110**	Piroxicam	**0**	1	1x	1	A	1	1	1
**111**	Pravastatin	**0**	0	0	0	A	0	0	0
**112**	Probenecid	**0**	0	0	0	A	0	0	0
**113**	Progesterone	**0**	0	0	0	A	0	0	0

**114**	Prostaglandin E2	**0**	0	0	0	A	0	0	0
**115**	Sulindac	**1**	1	1	1	A	1	1	1
**116**	Triiodo-L-thyronine (T3)	**1**	1	1	1x	A	1	1	1
**117**	β-Estradiol	**0**	0	0	0	A	0	0	0
**118**	Sulfobromophtalein	**1**	1x	1	1	A	1	1	1
**119**	Bilirubin	**1**	0	1	1	B	1	1	/
**120**	Biliverdin	**1**	1	1	1	A	1	1	1
	∑ active	50	54	49	58		53	42	30
	∑ predictions in AD	120	116	111	116		119	97	75

1 – inhibitor, 0 – noninhibitor, x – out of AD

Table S2: List of 300 phenolic compounds and their BTL activity predictions with separate models (NN-C, NN-D, Q-D) and consensus of models (A+B, NN-D+Q-D, A).

ID	Name	Ref.	NN-C	NN-D	Q-D	AB prediction	A+B	NN-D + Q-D	A
**FLAVONES**									
**1**	acacetin	[11]	1	1	0	B	1	/	/
**2**	chrysin	[80,87]	1	1	0	B	1	/	/
**3**	apigenin	[27,87]	1	1	0	B	1	/	/
**4**	luteolin	[27,87]	1	1	1	A	1	1	1
**5**	tangeretin	[27]	0x	0	0x	A	0	0	0
**6**	diosmetin	[27]	0	1	0	B	0	/	/
**7**	diosmin	[27]	1	0	0	B	0	0	/
**8**	baicalein	[27]	0	1	1	B	1	1	/
**9**	7,3 dimethylhesperetin	[27]	0	0	0	A	0	0	0
**10**	rhoifolin	[27]	0	0	0	A	0	0	0
**11**	flavone	[80]	0	1	0	B	0	/	/
**12**	3-methylflavone-8-carboxylic acid	[80]	1	1	0	B	1	/	/
**13**	flavonoid 1	[25]	0	1	0	B	0	/	/
**14**	flavonoid 2	[25]	0	1	0x	B	0	/	/
**15**	flavonoid 3	[25]	0	0	0x	A	0	0	0
**16**	flavonoid 4	[25]	0x	0	0x	A	0	0	0
**17**	flavonoid 5	[25]	0	0	0x	B	0	0	/
**18**	flavonoid 9	[25]	0	0	0	A	0	0	0
**19**	wogonin	[25]	0	1x	0	B	0	/	/
**20**	flavopiridol	[25]	0	0x	0	A	0	0	0
**21**	tricetin	[85]	1	0	1	B	1	/	/
**22**	isoetin	[85]	1	0	1	B	1	/	/
**23**	nortangeretin	[85]	1	0	1	B	1	/	/
**24**	6-fluroflavone	[21]	0	1	0	B	0	/	/
**25**	6-chloroflavone	[21]	0	1	0	B	0	/	/
**26**	6-bromoflavone	[21]	0	1	0	B	0	/	/
**27**	6-nitroflavone	[21]	0	1	0	B	0	/	/
**28**	6-methylflavone	[21]	0	1	0	B	0	/	/
**29**	3'-bromo-6-methylflavone	[21]	0	0	0	A	0	0	0
**30**	3'methyl-6-bromoflavone	[21]	0	1	0	B	0	/	/
**31**	3'-bromoflavone	[21]	0	1	0	B	0	/	/
**32**	3'-nitroflavone	[21]	0	1	0	B	0	/	/
**33**	4'-bromoflavone	[21]	0	1	0	B	0	/	/
**34**	3-bromoflavone	[21]	0	1	0	B	0	/	/
**35**	3,6-dibromoflavone	[21]	0	1	0	B	0	/	/
**36**	2'-nitroflavone	[21]	0	0	1	B	0	/	/
**37**	2',6-dinitroflavone	[21]	0	0	1	B	0	/	/
**38**	2',6-dinitro-3'-bromoflavone	[21]	0	1	1	B	1	1	/
**39**	2',6-difluoroflavone	[21]	0	1	0	B	0	/	/
**40**	2'-fluoro-6-chloroflavone	[21]	0	1	0	B	0	/	/
**41**	2'-fluoro-6-bromoflavone	[21]	0	1	0	B	0	/	/
**42**	5,7-dimethoxyflavone	[21]	0	1	0	B	0	/	/
**43**	α-naphthoflavone	[21]	0x	1x	0	B	0	/	/
**44**	β-naphthoflavone	[21]	0	1x	0	B	0	/	/
**45**	4'-nitroflavone	[28]	1	0	0	B	0	0	/

**46**	2'-chlorochrysin	[28]	0	1	0	B	0	/	/
**47**	kaempferitrin	[26]	1	0	0	B	0	0	/
**48**	kaempferol glycoside 2	[26]	0	0	0	A	0	0	0
**49**	kaempferol glycoside 3	[26]	1	0	0	B	0	0	/
**50**	kaempferol glycoside 4	[26]	0	0	0	A	0	0	0
**51**	6-hydroxyflavone	[84]	1	1	0	B	1	/	/
**52**	3'-hydroxyflavone	[84]	0	0	0	A	0	0	0
**53**	4'-hydroxyflavone	[84]	0	1x	0	B	0	/	/
**54**	kaempferol-3,4'-dimethylether	[84]	0	1	0	B	0	/	/
**55**	flavone-8-acetic acid (FAA)	[13]	0	1	0	B	0	/	/
**56**	FAAD-8	[13]	0x	0	1	B	0	/	/
**57**	FAAD-9	[13]	0	1x	0	B	0	/	/
**58**	FAAD-10	[13]	0	0x	0	A	0	0	0
**59**	FAAD-11	[13]	0	0x	1	B	0	/	/
**60**	FAAD-12	[13]	0	0x	1	B	0	/	/
**61**	FAAD-13	[13]	0	0x	1	B	0	/	/
**62**	FAAD-18b	[13]	0	1x	1	B	1	1	/
**63**	FAAD-19a	[13]	0	0x	1	B	0	/	/
**64**	FAAD-19b	[13]	0	0x	1	B	0	/	/
**65**	FAAD-19c	[13]	1	0x	1	B	1	/	/
**66**	FAAD-19d	[13]	0	0x	1	B	0	/	/
**67**	FAAD-19e	[13]	0	0x	1	B	0	/	/
**68**	FAAD-19f	[13]	0	0x	1	B	0	/	/
**69**	FAAD-19g	[13]	0	0x	1	B	0	/	/
**70**	FAAD-19h	[13]	1	0x	1	B	1	/	/
**71**	FAAD-19i	[13]	1	0x	1	B	1	/	/
**72**	FAAD-19j	[13]	0	0x	1	B	0	/	/
**73**	FAAD-19k	[13]	0	0x	1	B	0	/	/
**74**	FAAD-19l	[13]	0	0x	1	B	0	/	/
**75**	FAAD-19m	[13]	0	0x	0	A	0	0	0
**76**	FAAD-20	[13]	0	0x	1	B	0	/	/
**77**	FAAD-21	[13]	0	1x	1	B	1	1	/
**78**	FAAD-22	[13]	0	0x	1	B	0	/	/
**ISOFLAVONES**									
**79**	daidzin	[80]	1	0	0	B	0	0	/
**80**	daidzein	[80,87]	1	0	0	B	0	0	/
**81**	genistein	[27,87]	1	1	0	B	1	/	/
**82**	glycitein	[6]	1	1	0	B	1	/	/
**83**	genistin	[80]	1	0	0	B	0	0	/
**FLAVONOLS**									
**84**	galangin	[80,7]	1	1	0	B	1	/	/
**85**	tamarixetin	[80]	0	0	0	A	0	0	0
**86**	kaempferol	[80,87]	1	1	1	A	1	1	1
**87**	fisetin	[80]	0	1	0	B	0	/	/
**88**	3,6-dihydroxyflavone	[80]	1	1	0	B	1	/	/
**89**	morin	[17]	1	0	1	B	1	/	/
**90**	robinetin	[85]	1	0	0	B	0	0	/
**91**	herbacetin	[85]	0	0	1	B	0	/	/
**92**	3-hydroxyflavone	[84]	1	1	0	B	1	/	/
**93**	kaempferol-7-neohesperidoside	[84]	0	0	0	A	0	0	0
**FLAVANONES**									
**94**	pinocembrin	[6]	1	1	0	B	1	/	/
**95**	eriodictyol	[27,87]	1	1	1	A	1	1	1
**96**	Naringenin	[27,87]	1	1	0	B	1	/	/
**97**	Naringin	[27,87]	0	0	0	A	0	0	0
**98**	hesperidin	[87]	0	0	0	A	0	0	0
**99**	hesperetin	[27]	1	1	0	B	1	/	/
**100**	naringenoxy phthalonitrile	[83]	0x	0x	0	A	0	0	0
**101**	naringenoxy benzylphthalonitrile	[83]	1x	0x	0	B	0	0	/
**102**	2-naringenin-7-O-phthalocyaninatozinc	[83]	1	1x	0	B	1	/	/
**103**	luteolin glucoside	[87]	0	0	0	A	0	0	0
**104**	biochanin A	[8]	0	0	0	A	0	0	0
**105**	formononetin	[8]	0	0	0	A	0	0	0
**106**	flavonoid 6	[25]	0	0	0	A	0	0	0

**107**	flavonoid 7	[25]	0	0	0x	A	0	0	0
**108**	flavonoid 8	[25]	0	0	0x	A	0	0	0
**109**	flavonoid 10	[25]	1	0	0	B	0	0	/
**110**	flavanone	[21]	0x	0	0	A	0	0	0
**111**	6-hydroxyflavanone	[84]	0	0	0	A	0	0	0
**112**	2'-hydroxyflavanone	[84]	0	0x	0	A	0	0	0
**113**	3'-hydroxyflavanone	[84]	0	0	0	A	0	0	0
**114**	4'-hydroxyflavanone	[84]	0	0x	0	A	0	0	0
**115**	7'-hydroxyflavanone	[84]	0	0x	0	A	0	0	0
**116**	narigin	[84]	1	0	0	B	0	0	/
**FLAVANONOLS**									
**117**	pinobanksin	[6]	1	1	1	A	1	1	1
**118**	taxifolin	[17]	1	0	0	B	0	0	/
**119**	isorhamnetin	[8]	1	0	0	B	0	0	/
**120**	dihydrokaempferol (aromadendrin)	[85]	0	1	0	B	0	/	/
**121**	dihydromyrcetin (ampeloptin)	[85]	1	0	0	B	0	0	/
**122**	silymarin	[7]	1	0	0	B	0	0	/
**123**	fustin	[84]	0	1	0	B	0	/	/
**FLAVANES**									
**124**	epicatechin gallate	[87]	0	1	1	B	1	1	/
**125**	epigallocatechin gallate	[87]	0	1	1	B	1	1	/
**126**	theaflavin	[6]	0	1x	1	B	1	1	/
**127**	catechin (cianidanol)	[87]	1	1	1	A	1	1	1
**128**	gallocatechin	[87]	0	1	1	B	1	1	/
**FLAVANOLS**									
**129**	leucopelargonidin	[85]	0	1	1	B	1	1	/
**130**	leucocyanidin	[85]	0	1	1	B	1	1	/
**131**	leucodelphinidin	[85]	1	1	1	A	1	1	1
**132**	mangiferin	[6]	0	0	0	A	0	0	0
**XANTHONES**									
**133**	1,5-Dihydroxyxanthone	[6]	1	1	0	B	1	/	/
**134**	euxanthone	[6]	1	1	0	B	1	/	/
**CHROMONES**									
**135**	chromone	[80]	0	0x	0	A	0	0	0
**136**	7-hydroxy chromone	[80]	0	1x	0	B	0	/	/
**137**	3-formyl chromone	[80]	0	1x	0	B	0	/	/
**138**	chromone 2-acid carboxylic	[80]	1	1x	0	B	1	/	/
**139**	chromone 3-acid carboxylic	[80]	1	1x	0	B	1	/	/
**140**	2-amino-3-formylchromone	[80]	0x	1x	0	B	0	/	/
**141**	rohitukine	[25]	1	0x	0	B	0	0	/
**142**	CD-2a	[22]	0	1	0	B	0	/	/
**143**	CD-2b	[22]	0	0	0	A	0	0	0
**144**	CD-2c	[22]	0	1	0	B	0	/	/
**145**	CD-2d	[22]	0	1	0	B	0	/	/
**146**	CD-2e	[22]	0	1	1	B	1	1	/
**147**	CD-9a	[22]	0	0	1	B	0	/	/
**148**	CD-9b	[22]	0	0	0	A	0	0	0
**149**	CD-9c	[22]	0	0x	0	A	0	0	0
**150**	CD-9d	[22]	0	1	0	B	0	/	/
**151**	FAAD-23	[13]	0	1x	1	B	1	1	/
**152**	FAAD-24	[13]	1	1x	0	B	1	/	/
**153**	FAAD-28	[13]	0	1x	0	B	0	/	/
**154**	FAAD-29	[13]	1	1x	0	B	1	/	/
**COUMARINS**									
**155**	mammeigin	[6]	0	1x	0	B	0	/	/
**156**	mesuagin	[6]	0	1x	0	B	0	/	/
**157**	mammeisin	[6]	0	1x	0	B	0	/	/
**158**	mesuol	[6]	0	1x	0	B	0	/	/
**159**	CD-70	[82]	0	1	0	B	0	/	/
**160**	CD-71	[82]	0	0x	0	A	0	0	0
**161**	CD-76	[82]	0	1	0	B	0	/	/
**CHALCONES**									
**162**	OCD-1	[24]	0	0	0	A	0	0	0
**163**	OCD-2	[24]	0	0	0x	A	0	0	0

**164**	OCD-3	[24]	0	0	0x	A	0	0	0
**165**	OCD-4	[24]	0x	0	0x	A	0	0	0
**166**	OCD-5	[24]	1	1	0	B	1	/	/
**167**	OCD-6	[24]	0	0	0	A	0	0	0
**168**	OCD-7	[24]	1	1	0	B	1	/	/
**169**	OCD-8	[24]	0	0	0	A	0	0	0
**170**	OCD-9	[24]	0	0	0	A	0	0	0
**171**	OCD-10	[24]	0	0	0x	A	0	0	0
**PHENOLIC ACID DERIVATIVES**									
**172**	caffeic acid phenethy ester (CAPE)	[23]	0	1	0	B	0	/	/
**173**	caffeic acid (dihydroxycinnamic acid)	[30]	1	1	0	B	1	/	/
**174**	methyl salicylate	[6]	0	0	0	A	0	0	0
**175**	salicylic acid	[6]	0	0	0	A	0	0	0
**176**	L-DOPA	[6]	0	0	0	A	0	0	0
**177**	cichoric acid	[6]	0	1	0x	B	0	/	/
**178**	chlorogenic acid	[6]	0	0	1	B	0	/	/
**179**	cinnamic acid	[6]	0	0x	0	A	0	0	0
**180**	ferulic acid	[6]	0	1	0	B	0	/	/
**181**	ellagic acid	[6]	1	1	1	A	1	1	1
**182**	rosmarinic acid	[6]	1	1	1	A	1	1	1
**183**	methyl caffeate	[12]	0	1	0	B	0	/	/
**184**	ethyl caffeate	[12]	0	1	0	A	0	0	0
**185**	propyl caffeate	[12]	0	0x	0	A	0	0	0
**186**	isopropyl caffeate	[12]	0	0	0	A	0	0	0
**187**	butyl caffeate	[12]	0	0	0	A	0	0	0
**188**	octyl caffeate	[12]	0	0	0	A	0	0	0
**189**	dodecyl caffeate	[12]	0	0	0x	A	0	0	0
**190**	trimethoxybenzoic acid	[27]	0	0	0	A	0	0	0
**191**	sinapic acid	[27]	0	1	0	B	0	/	/
**192**	isoferulic	[27]	0	1	0	B	0	/	/
**193**	hexyl caffeate	[30]	0	0	0	A	0	0	0
**194**	hexyl ferulate	[30]	0	0	0	A	0	0	0
**195**	dihydrocaffeic acid	[86,19]	0	0	0	A	0	0	0
**196**	methyl dihydrocaffeate	[86]	0	0	0	A	0	0	0
**197**	ethyl dihydrocaffeate	[86]	0	0x	0	A	0	0	0
**198**	propily dihydrocaffeate	[86]	1	0x	0	B	0	0	/
**199**	gallic acid	[17]	1	1x	1	A	1	1	1
**200**	methyl gallate	[17]	1	1x	1	A	1	1	1
**201**	ethyl gallate	[17]	0	0	1	B	0	/	/
**202**	butyl gallate	[17]	0	0x	1	B	0	/	/
**203**	octyl gallate	[17]	0	0	1	B	0	/	/
**204**	syringic acid	[9]	0	1x	0	B	0	/	/
**205**	propyl gallate	[9]	0	0	1	B	0	/	/
**206**	dodecyl gallate	[9]	0	0	1x	B	0	/	/
**207**	DOPAC, 3,4-dihydroxyphenylacetic acid	[8,81]	0	1	0	B	0	/	/
**208**	HVA, 3-methoxy-4-hydroxyphenylacetic acid	[8,81]	1	0	0	B	0	0	/
**209**	3-methoxy-4-hydroxybenzoic acid	[8]	1	1x	1	A	1	1	1
**210**	protocatechuic acid	[8]	0	1x	1	B	1	1	/
**211**	3-methoxy-4-hydroxyhippuric acid	[8]	1	1x	1	A	1	1	1
**212**	caftaric acid	[6]	0	1	0x	B	0	/	/
**213**	chicoric acid	[6]	1	1	0x	B	1	/	/
**214**	phenylmethyl caffeate	[20]	0	1	0	B	0	/	/
**215**	phenylpropyl caffeate	[20]	0	1	0	B	0	/	/
**216**	phenylbutyl caffeate	[20]	0	1	0	B	0	/	/
**217**	phenylpentyl caffeate	[20]	0	1	0	B	0	/	/
**218**	phenylhexyl caffeate	[20]	0	1	0	B	0	/	/
**219**	phenyloctyl caffeate	[20]	0	1	0	B	0	/	/
**220**	phenyldodecanyl caffeate	[20]	0	1x	0	B	0	/	/
**221**	cinnamyl caffeate	[20]	0	1	0	B	0	/	/
**222**	8-phenyl-7-octenyl caffeate	[20]	0	1x	0	B	0	/	/
**223**	12-phenyl-11-dodecenyl caffeate	[20]	0	1x	0	B	0	/	/
**224**	2-cyclohexylethyl caffeate	[20]	0	0	0	A	0	0	0
**225**	decanyl caffeate	[20]	1	1x	1	A	1	1	1
**226**	oleuropein	[81]	1	1	1	A	1	1	1

**227**	3,4-DHPEA-EA	[81]	0	0	0	A	0	0	0
**228**	3,4-DHPEA-EDA	[81]	1	0	1	B	1	/	/
**229**	THPE - 2(3,4,5-trihydroxyphenyl)ethanoic acid	[19]	1	0	0	B	0	0	/
**230**	THPP - 3(3,4,5-trihydroxyphenyl)propanoic acid	[19]	0	1x	0	B	0	/	/
**231**	PAD2	[23]	0	1	0	B	0	/	/
**232**	PAD3	[23]	0	1	0	B	0	/	/
**233**	PAD4	[23]	0	1	0	B	0	/	/
**234**	PAD5	[23]	0	0	0	A	0	0	0
**235**	PAD6	[23]	0	0	0	A	0	0	0
**236**	PAD7	[23]	0	0	0	A	0	0	0
**237**	PAD8	[23]	0	1	0	B	0	/	/
**238**	PAD9	[23]	0	1	0	B	0	/	/
**239**	PAD10	[23]	0	1	0	B	0	/	/
**240**	PAD11	[23]	0	1	0	B	0	/	/
**241**	PAD12	[23]	0	1	0	B	0	/	/
**242**	PAD13	[23]	0	1	0	B	0	/	/
**243**	PAD14	[23]	1	1	0	B	1	/	/
**SIMPLE PHENOLS**									
**244**	carvacrol	[6]	0	0	0	A	0	0	0
**245**	eugenol	[6]	0	0	0	A	0	0	0
**246**	guaiacol	[6]	0x	0	0	A	0	0	0
**247**	rheosmin (raspberry ketone)	[6]	0	0	0	A	0	0	0
**248**	butylated hydroxytoluene	[6]	0x	0	1x	B	0	/	/
**249**	2,6-xylenol	[6]	0	0	0	A	0	0	0
**250**	propofol (Diprivan)	[6]	0x	0	1x	B	0	/	/
**251**	butylhydroquinone	[18]	0	0x	1	B	0	/	/
**252**	3,5-dibutylcatechol	[18]	0	0x	0	A	0	0	0
**253**	gingerol	[6]	1	0	0	B	0	0	/
**254**	tyrosol	[81]	0	0x	0	A	0	0	0
**255**	hydroxytyrosol	[81]	0	0x	0	A	0	0	0
**256**	MOPET	[81]	0	0	0	A	0	0	0
**257**	tyramine	[6]	0	0	0	A	0	0	0
**OTHERS**									
**258**	α-tocopherol (vitamin E)	[86]	0x	0	0x	A	0	0	0
**259**	retinol (vitamin A)	[6]	0	0	0x	A	0	0	0
**260**	pterostilbene	[6]	0	0	0	A	0	0	0
**261**	silibinin	[6]	1	0	0	B	0	0	/
**262**	capsaicin	[6]	0x	0	0	A	0	0	0
**263**	cannabidiol	[6]	0	1x	0	B	0	/	/
**264**	thymol	[6]	0	0	0	A	0	0	0
**265**	sesamol	[6]	0x	0	0	A	0	0	0
**266**	sitosterol	[6]	0x	0x	0	A	0	0	0
**267**	bisphenol A (BPA)	[6]	0	0x	1x	B	0	/	/
**268**	2-phenylphenol	[6]	0x	0	0	A	0	0	0
**269**	caffeoylhexylamide	[30]	0	0x	0	A	0	0	0
**270**	feruloylhexylamide	[30]	1	0	0	B	0	0	/
**271**	apigenidin	[87]	0	1	0	B	0	/	/
**272**	caffeine	[7]	0x	0x	0	A	0	0	0
**273**	tamoxifen	[24]	0	0x	0	A	0	0	0
**274**	paradol	[12]	1	0	0	B	0	0	/
**275**	PAD15	[23]	0	1	0	B	0	/	/
**276**	PAD16	[23]	0	1	0	B	0	/	/
**277**	PAD17	[23]	0	1	0	B	0	/	/
**278**	PACD1	[88]	0	0x	1	B	0	/	/
**279**	PACD2	[88]	1	1x	0	B	1	/	/
**280**	PACD3	[88]	1	1x	1	A	1	1	1
**281**	PACD4	[88]	0	0x	0	A	0	0	0
**282**	PACD5	[88]	0	0x	0	A	0	0	0
**283**	PACD6	[88]	0	0	1	B	0	/	/
**284**	PACD7	[88]	0	0x	0	A	0	0	0
**285**	PACD8	[88]	0	1x	1	B	1	1	/
**286**	PACD9	[88]	0	0x	0	A	0	0	0
**287**	PACD10	[88]	0	0x	0	A	0	0	0
**288**	PACD11	[88]	1	0	0	B	0	0	/

**289**	PACD12	[88]	0	1x	0	B	0	/	/
**290**	PACD13	[88]	1	0x	0	B	0	0	/
**291**	PACD14	[88]	0	0x	1	B	0	/	/
**292**	PACD15	[88]	0	0	1	B	0	/	/
**293**	PACD16	[88]	1	1	0	B	1	/	/
**294**	PACD17	[88]	0	0x	0	A	0	0	0
**295**	PACD18	[88]	0	1	1	B	1	1	/
**296**	PACD19	[88]	0	0	0x	A	0	0	0
**297**	PACD20	[88]	0	1	0	B	0	/	/
**298**	PACD21	[88]	0	0x	0	A	0	0	0
**299**	gelastatin hydroxamate	[22]	0	0	0	A	0	0	0
**300**	bestatin	[13]	0	0x	1	B	0	/	/
		∑ active	75	138	72	/	65	31	15
		∑ predictions in AD	283	208	278	/	300	151	109

1 – inhibitor, 0 – noninhibitor, x – out of AD

Table S3: List of predictive QSAR models of 14 membrane transporters available at ChemBench platform.

Model	ID	Description	no. objects	ACC	CCR	SP	SE	Descriptors
**MDR1**	314a	Multidrug resistance protein 1 (MDR1, ABCB1, P-gp) inhibition at 10uM	1585	0.91	0.91	0.91	0.91	Dragon X-H
	313a	Multidrug resistance protein 1 (MDR1, ABCB1, P-gp) substrates vs. non-substrates	567	0.74	0.74	0.68	0.80	Dragon X-H
**BSEP**	243x	Bile Salt Export Pump (BSEP, ABCB11) inhibition at 10uM	725	0.84	0.84	0.87	0.80	Dragon X-H
	242x	Bile Salt Export Pump (BSEP, ABCB11) substrates vs. non-substrates	34	0.68	0.68	0.68	0.69	Dragon X-H
**BCRP**	241x	Breast cancer resistance protein (BCRP, ABCG2) inhibition at 10uM	395	0.83	0.82	0.87	0.77	Dragon X-H
	234q	Breast cancer resistance protein (BCRP, ABCG2) substrates vs. non-substrates	169	0.79	0.79	0.71	0.86	CDK
**MRP1**	322z	Multidrug resistance-associated protein 1 (MRP1, ABCC1) inhibition at 10uM	426	0.84	0.84	0.84	0.84	CDK
	321x	Multidrug resistance-associated protein 1 (MRP1, ABCC1) substrates vs. non-substrates	180	0.86	0.86	0.86	0.86	Dragon X-H
**MRP2**	324s_RF	Multidrug resistance-associated protein 2 (MRP2, ABCC2) inhibition at 10uM	104	0.83	0.83	0.79	0.87	Dragon X-H
	323x	Multidrug resistance-associated protein 2 (MRP2, ABCC2) substrates vs. non-substrates	222	0.80	0.80	0.79	0.81	Dragon X-H
**MRP3**	331x	Multidrug resistance-associated protein 3 (MRP3, ABCC3) inhibition at 10uM	36	0.64	0.62	0.74	0.53	Dragon X-H
	333a	Multidrug resistance-associated protein 3 (MRP3, ABCC3) substrate vs. non-substrates	100	0.92	0.92	0.94	0.90	Dragon X-H
**MRP4**	334q	Multidrug resistance-associated protein 4 (MRP4, ABCC4) inhibition at 10uM	67	0.78	0.78	0.78	0.77	CDK
	342z	Multidrug resistance-associated protein 4 (MRP4, ABCC4) substrates vs. non-substrates	122	0.76	0.76	0.74	0.79	CDK
**MRP5**	343z	Multidrug resistance-associated protein 5 (MRP5, ABCC5) inhibition at 50uM	37	0.84	0.79	0.62	0.96	CDK
	344q	Multidrug resistance-associated protein 5 (MRP5, ABCC5) substrates vs. non-substrates	58	0.76	0.76	0.76	0.76	CDK
**MCT1**	312z	Monocarboxylate transporter 1 (MCT1, SLC16A1) inhibition at 10uM	68	0.96	0.95	0.95	0.96	CDK
	311x	Monocarboxylate transporter 1 (MCT1, SLC16A1) substrates vs. non-substrates	36	0.78	0.78	0.89	0.67	Dragon X-H
**NTCP**	411x	Na+-taurocholate cotransporting polypeptide (NTCP, LBAT, SLC10A1) inhibition at 10uM	127	0.81	0.80	0.86	0.74	Dragon X-H
	412q	Na+-taurocholate cotransporting polypeptide (NTCP, LBAT, SLC10A1) substrates vs. non-substrates	70	0.89	0.89	0.87	0.89	CDK
**ASBT**	232z	Apical sodium-dependent bile acid transporter (ASBT, IBAT, SLC10A2) inhibition at 10uM	232	0.89	0.89	0.85	0.92	CDK
	233f_RF	Apical sodium-dependent bile acid transporter (ASBT, IBAT, SLC10A2) substrates vs. non-substrates	106	0.89	0.89	0.83	0.94	Dragon X-H
**OCT1**	421z	Organic cation transporter 1 (OCT1, SLC22A1) inhibition at 100uM	199	0.87	0.86	0.92	0.81	CDK
	422z	Organic cation transporter 1 (OCT1, SLC22A1) substrates vs. non-substrates	82	0.87	0.87	0.88	0.85	CDK

**OATP2B1**	414z	Organic anion transporting polypeptide 2B1 (OATP2B1, SLCO2B1, SLC21A9) inhibition at 100uM	114	0.83	0.83	0.79	0.88	CDK
	413x	Organic anion transporting polypeptide 2B1 (OATP2B1, SLCO2B1, SLC21A9) substrates vs. non-substrates	59	0.81	0.81	0.77	0.85	Dragon X-H
**PEPT1**	423z	Peptide transporter 1 (PEPT1, SLC15A1) inhibition at 100uM	82	0.74	0.74	0.73	0.76	CDK
	424z	Peptide transporter 1 (PEPT1, SLC15A1) substrates vs. non-substrates	292	0.86	0.86	0.83	0.89	CDK
**BTL***	NN-2	Bilirubin membrane transporter (Bilitranslocase) inhibition	120	0.91	0.91	0.93	0.88	Dragon X-H

*new, not available in ChemBench, CCR=0.5 SE + 0.5 SP

Table S4: List of selected Dragon descriptors in NN-D and Q-D models.

NN-D model	Q-D model
nBM	ARR
nO	LOC
nP	VE3sign_Dz(p)
VE1_A	EE_B(s)
MATS7e	P_VSA_p_4
GATS5i	nArCO
P_VSA_LogP_2	nArOR
nArCO	H-052
nOHp	CATS2D_07_DA
nSO3OH	CATS2D_04_AN
nP(=O)O2R	B05[O-O]
SsssN	
CATS2D_03_LL	
T(Cl,,Cl)	
B03[N-O]	
B04[O-S]	
DLS_05	
LLS_01	

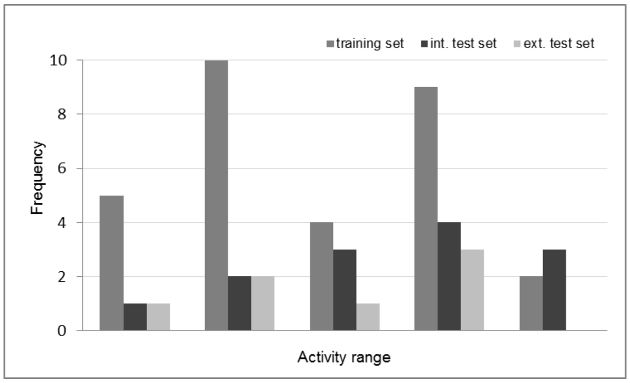

Figure S1: Frequency distribution of compounds for five ranges of BTL biological activity (< 2.5, 2.5 - 3.5, 3.5 – 4.5, 4.5 – 5.5, > 5.5) in datasets used for model building and validation.

Table S5: Hamming distances among the best four BTL regression models (M1-M4).

	M2	M3	M4
**M1**	8	15	9
**M2**	-	17	7
**M3**	-	-	10

Table S6: Summary of new compounds predictions with the best four BTL regression models.

		No. of compounds in the AD		
**Model**	No. of descriptors	Phenols ∑ = 205	Antioxidants* ∑ = 29	Antiprions* ∑ = 19
**M1**	12	152	0	2
**M2**	10	157	17	3
**M3**	9	97	5	1
**M4**	5	183	20	2

*results from study of Martinčič *et al.* [59]

Table S7: BTL activity predictions with regression models M1-M4 for phenolic compounds, which were classified as active in classification models. ID is the same as in Table S2.

ID	Active in classification models	M1	M2	M3	M4	$\bar{y} \pm SD$	$\bar{y}_w$
**1**	NN-C, NN-D	4.44	4.21	4.20	4.21	**4.27 ± 0.12**	**4.24**
**2**	NN-C, NN-D	4.16	4.15	3.98	4.21	**4.13 ± 0.10**	**4.14**
**3**	NN-C, NN-D	4.16	4.15	3.98	4.06	**4.09 ± 0.08**	**4.10**
**4**	NN-C, NN-D, Q-D	4.71	3.86	4.20	4.06	**4.21 ± 0.36**	**4.16**
**6**	NN-D	4.44	4.21	4.20	4.06	**4.23 ± 0.16**	**4.21**
**7**	NN-C	4.88	5.52	4.72	5.04	**5.04 ± 0.35**	**5.04**
**8**	NN-D, Q-D	3.94	4.15	4.20	4.06	**4.09 ± 0.12**	**4.09**
**11x**	NN-D	4.16	4.15	3.98	4.21	**4.13 ± 0.10**	**4.13**
**12**	NN-C, NN-D	4.16	4.15	3.98	4.21	**4.13 ± 0.10**	**4.14**
**13**	NN-D	4.03	4.15	3.98	4.06	**4.06 ± 0.07**	**4.05**
**14**	NN-D	4.16	4.15	3.98	4.21	**4.13 ± 0.10**	**4.12**
**19**	NN-D	4.16	4.15	3.98	4.06	**4.09 ± 0.08**	**4.07**
**21**	NN-C, Q-D	4.71	4.70	4.66	4.71	**4.70 ± 0.02**	**4.70**
**22**	NN-C, Q-D	4.71	4.70	4.66	4.71	**4.70 ± 0.02**	**4.70**
**23**	NN-C, Q-D	4.71	4.70	4.66	3.80	**4.47 ± 0.45**	**4.38**
**24**	NN-D	4.16	4.15	3.98	4.21	**4.13 ± 0.10**	**4.13**
**25**	NN-D	4.16	4.15	3.98	4.21	**4.13 ± 0.10**	**4.12**
**26**	NN-D	4.16	4.15	3.98	4.21	**4.13 ± 0.10**	**4.12**
**27**	NN-D	4.16	4.15	3.98	4.21	**4.13 ± 0.10**	**4.12**
**28**	NN-D	4.16	4.15	3.98	4.21	**4.13 ± 0.10**	**4.12**
**30**	NN-D	4.00	4.15	3.98	4.21	**4.08 ± 0.11**	**4.09**
**31**	NN-D	4.16	4.15	3.98	4.21	**4.13 ± 0.10**	**4.12**
**32**	NN-D	4.16	4.15	3.98	4.21	**4.13 ± 0.10**	**4.12**
**33**	NN-D	4.16	4.15	3.98	4.21	**4.13 ± 0.10**	**4.12**
**34x**	NN-D	4.16	4.15	3.98	4.21	**4.13 ± 0.10**	**4.13**
**35**	NN-D	4.16	4.15	3.98	4.21	**4.13 ± 0.10**	**4.12**
**36**	Q-D	4.16	4.15	3.98	4.21	**4.13 ± 0.10**	**4.12**
**37x**	Q-D	4.16	4.15	3.98	4.21	**4.13 ± 0.10**	**4.12**
**38x**	NN-D,Q-D	4.00	3.78	3.98	4.21	**3.99 ± 0.17**	**4.02**

**39**	NN-D, Q-D	4.16	4.15	3.98	4.21	**4.13**	±	**0.10**	**4.13**
**40x**	NN-D, Q-D	4.16	4.15	3.98	4.21	**4.13**	±	**0.10**	**4.13**
**41x**	NN-D, Q-D	4.16	4.15	3.98	4.21	**4.13**	±	**0.10**	**4.12**
**42**	NN-D, Q-D	4.16	4.15	3.98	4.21	**4.13**	±	**0.10**	**4.14**
**43x**	NN-D	4.16	4.15	3.98	4.21	**4.13**	±	**0.10**	**4.14**
**44x**	NN-D	4.16	4.15	3.98	4.21	**4.13**	±	**0.10**	**4.14**
**45**	NN-C	4.16	4.15	3.98	4.21	**4.13**	±	**0.10**	**4.12**
**46**	NN-D	4.03	4.15	3.98	4.21	**4.09**	±	**0.10**	**4.12**
**47**	NN-C	4.81	5.52	5.48	5.04	**5.21**	±	**0.35**	**5.19**
**49**	NN-C	5.20	5.20	5.20	5.20	**5.20**	±	**0.00**	**5.20**
**51**	NN-C, NN-D	4.16	4.15	3.98	4.21	**4.13**	±	**0.10**	**4.13**
**53**	NN-D	4.16	4.15	3.98	4.21	**4.13**	±	**0.10**	**4.13**
**54**	NN-D	4.16	4.15	3.98	4.21	**4.13**	±	**0.10**	**4.14**
**55**	NN-D	4.16	4.15	3.98	4.21	**4.13**	±	**0.10**	**4.11**
**56**	Q-D	4.00	4.21	3.98	3.91	**4.03**	±	**0.13**	**4.01**
**57**	NN-D	4.16	4.15	3.98	3.91	**4.05**	±	**0.12**	**4.05**
**59x**	Q-D	4.16	4.15	3.98	4.21	**4.13**	±	**0.10**	**4.12**
**60**	Q-D	4.16	4.15	3.98	3.82	**4.03**	±	**0.16**	**4.03**
**61**	Q-D	4.16	4.15	3.98	3.82	**4.03**	±	**0.16**	**4.01**
**62x**	NN-D, Q-D	4.00	4.15	3.98	4.21	**4.08**	±	**0.11**	**4.08**
**63**	NN-D	4.16	4.15	3.98	3.82	**4.03**	±	**0.16**	**4.02**
**64**	NN-D	4.00	3.78	3.98	3.82	**3.90**	±	**0.11**	**3.89**
**65**	NN-C, Q-D	4.00	3.78	3.98	4.88	**4.16**	±	**0.49**	**4.27**
**66**	Q-D	4.00	3.78	3.98	3.82	**3.90**	±	**0.11**	**3.88**
**67**	Q-D	4.00	3.78	3.98	3.82	**3.90**	±	**0.11**	**3.88**
**68**	Q-D	4.00	3.78	3.98	3.82	**3.90**	±	**0.11**	**3.89**
**69**	Q-D	4.00	3.78	3.98	3.82	**3.90**	±	**0.11**	**3.89**
**70**	NN-C, Q-D	4.00	3.78	3.98	3.82	**3.90**	±	**0.11**	**3.88**
**71**	NN-C, Q-D	4.00	3.78	3.98	4.88	**4.16**	±	**0.49**	**4.22**
**72**	Q-D	4.00	4.21	3.98	4.88	**4.27**	±	**0.42**	**4.28**
**73**	Q-D	4.00	3.78	3.98	4.88	**4.16**	±	**0.49**	**4.24**
**74**	Q-D	4.00	3.78	3.98	4.88	**4.16**	±	**0.49**	**4.24**
**76**	Q-D	4.16	3.78	3.98	4.88	**4.20**	±	**0.48**	**4.35**
**77x**	NN-D, Q-D	4.00	3.78	5.23	4.82	**4.45**	±	**0.68**	**4.57**
**78**	Q-D	2.30	4.21	3.98	3.91	**3.60**	±	**0.87**	**3.72**
**79**	NN-C	4.62	4.61	5.47	4.06	**4.69**	±	**0.58**	**4.62**
**80**	NN-C	4.16	4.15	3.98	4.21	**4.13**	±	**0.10**	**4.14**
**81**	NN-C, NN-D	4.16	4.15	3.98	4.21	**4.13**	±	**0.10**	**4.14**
**82**	NN-C, NN-D	4.44	3.86	4.20	4.21	**4.18**	±	**0.24**	**4.18**
**83**	NN-C	5.14	4.61	5.47	3.85	**4.77**	±	**0.71**	**4.69**
**84**	NN-C, NN-D	4.16	4.15	4.20	4.21	**4.18**	±	**0.03**	**4.18**
**86**	NN-C, NN-D, Q-D	3.94	4.15	4.20	4.21	**4.12**	±	**0.13**	**4.12**
**87**	NN-D	3.96	3.86	4.66	3.80	**4.07**	±	**0.40**	**4.16**
**88**	NN-C, NN-D	4.16	4.15	3.98	4.21	**4.13**	±	**0.10**	**4.14**
**89**	NN-C, Q-D	4.71	4.70	4.66	4.71	**4.70**	±	**0.02**	**4.69**
**90**	NN-C	4.74	4.99	4.66	5.05	**4.86**	±	**0.19**	**4.86**
**91**	Q-D	4.71	4.70	4.66	4.71	**4.70**	±	**0.02**	**4.68**
**92**	NN-C, NN-D	4.16	4.15	3.98	4.21	**4.13**	±	**0.10**	**4.14**
**94**	NN-C, NN-D	4.16	4.15	3.98	4.21	**4.13**	±	**0.10**	**4.10**
**95**	NN-C, NN-D, Q-D	5.19	4.96	4.66	2.43	**4.31**	±	**1.27**	**4.30**
**96**	NN-C, NN-D	3.94	4.21	4.20	3.18	**3.88**	±	**0.48**	**3.90**
**99**	NN-C, NN-D	2.30	4.21	3.98	3.18	**3.42**	±	**0.86**	**3.39**
**101x**	NN-C	4.16	3.78	5.42	4.82	**4.54**	±	**0.72**	**4.65**
**102x**	NN-C, NN-D	5.45	5.45	5.46	5.46	**5.46**	±	**0.01**	**5.46**
**109**	NN-C	4.16	4.15	3.98	4.88	**4.30**	±	**0.40**	**4.34**
**116**	NN-C	5.25	5.52	4.72	5.04	**5.13**	±	**0.34**	**5.14**
**117**	NN-C, NN-D, Q-D	4.44	4.72	4.20	3.18	**4.14**	±	**0.67**	**4.13**
**118**	NN-C	5.19	5.25	4.66	4.06	**4.79**	±	**0.55**	**4.89**
**119**	NN-C	2.30	2.30	4.20	4.06	**3.22**	±	**1.06**	**3.11**
**120**	Q-D	5.19	5.25	4.66	4.06	**4.79**	±	**0.55**	**4.90**
**121**	NN-C	5.24	5.11	5.28	3.56	**4.80**	±	**0.83**	**4.71**
**122**	NN-C	5.68	5.70	5.23	5.62	**5.56**	±	**0.22**	**5.56**
**123**	NN-D	4.79	5.25	4.66	4.06	**4.69**	±	**0.49**	**4.86**
**124**	NN-D, Q-D	4.62	4.61	5.23	3.25	**4.43**	±	**0.84**	**4.18**

**125**	NN-D, Q-D	4.62	4.61	5.23	3.06	**4.38**	±	**0.92**	**4.03**
**126**	NN-D, Q-D	5.50	5.52	5.47	5.35	**5.46**	±	**0.08**	**5.44**
**127x**	NN-C, NN-D, Q-D	4.79	5.25	5.13	2.43	**4.40**	±	**1.32**	**4.40**
**128x**	NN-D, Q-D	5.19	5.25	5.13	4.64	**5.05**	±	**0.28**	**5.05**
**129**	NN-D, Q-D	5.19	5.25	5.13	2.43	**4.50**	±	**1.38**	**4.50**
**130x**	NN-D, Q-D	5.19	4.93	5.13	4.64	**4.97**	±	**0.25**	**4.96**
**131x**	NN-C, NN-D, Q-D	5.24	5.11	5.13	2.95	**4.61**	±	**1.11**	**4.48**
**133**	NN-C, NN-D	4.16	4.15	3.98	4.21	**4.13**	±	**0.10**	**4.14**
**134**	NN-C, NN-D	4.16	4.15	3.98	4.21	**4.13**	±	**0.10**	**4.14**
**136x**	NN-D	4.00	4.24	4.25	4.21	**4.17**	±	**0.12**	**4.19**
**137x**	NN-D	4.16	4.15	4.23	4.21	**4.19**	±	**0.04**	**4.19**
**138x**	NN-C, NN-D	4.16	4.15	4.23	4.21	**4.19**	±	**0.04**	**4.19**
**139x**	NN-C, NN-D	4.16	4.15	4.23	4.21	**4.19**	±	**0.04**	**4.19**
**140**	NN-D	3.94	3.59	4.23	3.80	**3.89**	±	**0.27**	**3.91**
**141**	NN-C	2.30	2.30	3.69	3.91	**3.05**	±	**0.87**	**3.17**
**142**	NN-D	4.03	4.24	3.27	4.21	**3.94**	±	**0.45**	**3.90**
**144**	NN-D	4.00	4.24	3.27	4.21	**3.93**	±	**0.45**	**3.91**
**145**	NN-D	4.00	4.24	3.27	4.21	**3.93**	±	**0.45**	**3.91**
**146**	NN-D, Q-D	3.50	3.59	2.21	3.91	**3.30**	±	**0.75**	**3.31**
**147**	Q-D	3.50	3.59	2.21	3.82	**3.28**	±	**0.73**	**3.29**
**150**	NN-D	4.00	3.78	3.98	3.82	**3.90**	±	**0.11**	**3.89**
**151x**	NN-D, Q-D	4.00	3.78	3.98	4.88	**4.16**	±	**0.49**	**4.25**
**152**	NN-C, NN-D	4.03	3.86	4.20	4.88	**4.24**	±	**0.45**	**4.28**
**153**	NN-D	4.03	3.86	4.20	4.73	**4.21**	±	**0.38**	**4.21**
**154**	NN-C, NN-D	4.44	3.86	4.66	4.73	**4.42**	±	**0.40**	**4.41**
**155x**	NN-D	4.00	4.15	3.98	4.88	**4.25**	±	**0.43**	**4.38**
**156**	NN-D	4.16	4.15	3.98	3.82	**4.03**	±	**0.16**	**4.00**
**157x**	NN-D	3.50	3.78	5.47	4.88	**4.41**	±	**0.93**	**4.51**
**158x**	NN-D	3.50	3.78	3.98	3.82	**3.77**	±	**0.20**	**3.79**
**159**	NN-D	4.00	3.78	3.98	4.21	**3.99**	±	**0.17**	**4.03**
**161**	NN-D	4.00	3.78	3.98	3.82	**3.90**	±	**0.11**	**3.89**
**166**	NN-C, NN-D	4.16	4.15	4.20	4.21	**4.18**	±	**0.03**	**4.18**
**168**	NN-C, NN-D	4.44	3.86	4.66	3.85	**4.20**	±	**0.41**	**4.24**
**172**	NN-D	4.16	4.21	3.98	4.21	**4.14**	±	**0.11**	**4.13**
**173**	NN-C, NN-D	3.94	3.86	4.23	3.80	**3.96**	±	**0.19**	**3.96**
**177**	NN-D	5.46	5.87	5.23	3.85	**5.10**	±	**0.88**	**4.82**
**178**	Q-D	4.62	5.11	5.13	5.22	**5.02**	±	**0.27**	**5.01**
**180**	NN-D	4.03	4.24	4.23	4.06	**4.14**	±	**0.11**	**4.13**
**181**	NN-C, NN-D, Q-D	3.96	3.59	4.66	4.25	**4.12**	±	**0.45**	**4.13**
**182**	NN-C, NN-D, Q-D	5.24	4.23	4.66	3.85	**4.50**	±	**0.60**	**4.52**
**183**	NN-D	3.94	4.15	4.23	4.21	**4.13**	±	**0.13**	**4.16**
**184**	NN-D	4.03	4.21	3.27	4.21	**3.93**	±	**0.45**	**3.97**
**191**	NN-D	4.16	4.15	3.98	4.21	**4.13**	±	**0.10**	**4.15**
**192**	NN-D	4.03	4.24	4.23	4.06	**4.14**	±	**0.11**	**4.13**
**198**	NN-C	4.03	4.21	2.21	4.21	**3.66**	±	**0.97**	**3.64**
**199**	NN-C, NN-D, Q-D	3.49	2.89	3.79	4.25	**3.61**	±	**0.57**	**3.67**
**200**	NN-C, NN-D, Q-D	3.49	3.59	3.79	3.80	**3.67**	±	**0.15**	**3.66**
**201**	Q-D	3.94	3.59	3.79	3.80	**3.78**	±	**0.14**	**3.78**
**202**	Q-D	3.94	3.86	5.08	3.80	**4.17**	±	**0.61**	**4.10**
**203**	Q-D	4.16	4.21	5.08	4.21	**4.42**	±	**0.45**	**4.32**
**204**	NN-D	4.16	4.15	3.79	4.21	**4.08**	±	**0.19**	**4.11**
**205**	Q-D	3.94	3.86	3.79	3.80	**3.85**	±	**0.07**	**3.85**
**206x**	Q-D	4.16	5.87	5.95	4.88	**5.22**	±	**0.85**	**5.05**
**207**	NN-D	3.94	3.59	4.73	3.80	**4.02**	±	**0.50**	**4.13**
**208**	NN-C	4.03	3.59	4.23	4.21	**4.01**	±	**0.29**	**4.08**
**209**	NN-C, NN-D, Q-D	3.94	3.59	4.23	4.06	**3.96**	±	**0.27**	**4.00**
**210**	NN-D, Q-D	3.49	3.59	3.79	4.25	**3.78**	±	**0.34**	**3.79**
**211**	NN-C, NN-D, Q-D	2.30	3.59	3.27	3.18	**3.09**	±	**0.55**	**3.11**
**212**	NN-D	3.32	4.23	3.79	3.85	**3.80**	±	**0.38**	**3.75**
**213**	NN-C, NN-D	5.46	5.87	5.23	3.85	**5.10**	±	**0.88**	**4.70**
**214**	NN-D	4.16	4.15	3.98	4.21	**4.13**	±	**0.10**	**4.12**
**215**	NN-D	4.16	3.78	3.98	4.21	**4.03**	±	**0.20**	**4.07**
**216**	NN-D	4.16	3.78	3.98	4.21	**4.03**	±	**0.20**	**4.08**
**217**	NN-D	4.16	3.78	3.98	4.21	**4.03**	±	**0.20**	**4.09**

**218**	NN-D	4.16	3.78	3.98	4.21	**4.03**	±	**0.20**	**4.10**
**219**	NN-D	4.16	3.78	5.47	4.88	**4.57**	±	**0.75**	**4.69**
**220x**	NN-D	4.16	5.87	5.86	4.88	**5.20**	±	**0.83**	**5.01**
**221**	NN-D	4.16	3.78	3.98	4.21	**4.03**	±	**0.20**	**4.11**
**222**	NN-D	4.16	3.78	5.47	4.88	**4.57**	±	**0.75**	**4.70**
**223x**	NN-D	4.16	5.87	5.23	4.88	**5.04**	±	**0.71**	**4.92**
**225**	NN-C, NN-D, Q-D	4.16	3.78	5.85	4.21	**4.50**	±	**0.92**	**4.37**
**226**	NN-C, NN-D, Q-D	4.34	4.32	4.23	4.38	**4.32**	±	**0.06**	**4.32**
**228**	NN-C, Q-D	2.30	3.78	3.69	3.18	**3.24**	±	**0.68**	**3.22**
**229**	NN-C	3.82	3.59	5.08	5.05	**4.39**	±	**0.79**	**4.48**
**230**	NN-D	3.96	3.86	5.08	5.05	**4.49**	±	**0.67**	**4.59**
**231**	NN-D	4.16	3.78	3.98	4.21	**4.03**	±	**0.20**	**4.06**
**232**	NN-D	4.16	3.78	3.98	4.21	**4.03**	±	**0.20**	**4.05**
**233**	NN-D	4.16	4.15	3.98	4.21	**4.13**	±	**0.10**	**4.12**
**237**	NN-D	4.44	4.21	4.20	4.21	**4.27**	±	**0.12**	**4.25**
**238**	NN-D	3.50	3.78	3.98	4.21	**3.87**	±	**0.30**	**3.93**
**239**	NN-D	4.03	3.78	3.98	4.21	**4.00**	±	**0.18**	**4.03**
**240**	NN-D	4.16	3.78	3.98	4.21	**4.03**	±	**0.20**	**4.06**
**241**	NN-D	4.16	4.21	3.98	4.21	**4.14**	±	**0.11**	**4.14**
**242**	NN-D	4.03	3.78	3.98	4.21	**4.00**	±	**0.18**	**4.03**
**243**	NN-C, NN-D	4.16	3.78	3.98	4.21	**4.03**	±	**0.20**	**4.06**
**248x**	Q-D	4.16	4.15	5.28	2.43	**4.01**	±	**1.17**	**3.81**
**250x**	Q-D	4.16	4.15	5.28	2.43	**4.01**	±	**1.17**	**3.90**
**251x**	Q-D	4.16	2.30	2.21	2.43	**2.78**	±	**0.93**	**2.73**
**253**	NN-C	2.30	3.78	3.71	2.43	**3.06**	±	**0.80**	**2.93**
**261**	NN-C	5.68	5.70	5.47	5.62	**5.62**	±	**0.11**	**5.62**
**263x**	NN-D	3.50	3.78	5.74	3.18	**4.05**	±	**1.15**	**4.02**
**267x**	Q-D	4.16	4.15	3.98	2.43	**3.68**	±	**0.84**	**3.59**
**270**	NN-C	3.50	3.78	2.48	4.21	**3.49**	±	**0.73**	**3.74**
**271**	NN-D	4.16	3.95	4.20	3.74	**4.01**	±	**0.21**	**4.02**
**274x**	NN-C	4.16	3.78	3.98	4.21	**4.03**	±	**0.20**	**4.07**
**275**	NN-D	4.74	4.96	4.66	3.80	**4.54**	±	**0.51**	**4.49**
**276**	NN-D	3.87	3.78	4.20	3.91	**3.94**	±	**0.18**	**3.95**
**277**	NN-D	3.87	3.78	4.20	3.91	**3.94**	±	**0.18**	**3.95**
**278x**	Q-D	4.00	4.15	3.98	4.21	**4.08**	±	**0.11**	**4.10**
**279x**	NN-C, NN-D	3.50	3.86	3.79	4.88	**4.01**	±	**0.60**	**4.06**
**280**	NN-C, NN-D, Q-D	5.14	4.23	4.79	3.85	**4.50**	±	**0.58**	**4.32**
**283**	Q-D	4.03	3.86	2.48	3.91	**3.57**	±	**0.73**	**3.65**
**285**	NN-D, Q-D	4.03	4.21	3.71	3.82	**3.94**	±	**0.22**	**3.98**
**288**	NN-C	4.00	4.15	4.23	4.21	**4.15**	±	**0.10**	**4.16**
**289x**	NN-D	3.32	3.86	3.78	4.88	**3.96**	±	**0.66**	**4.03**
**290**	NN-C	3.32	2.44	3.78	2.53	**3.02**	±	**0.65**	**2.91**
**291**	Q-D	2.99	3.38	3.71	3.84	**3.48**	±	**0.38**	**3.56**
**292x**	Q-D	4.16	4.15	4.71	4.88	**4.48**	±	**0.38**	**4.51**
**293**	NN-C, NN-D	3.50	4.15	2.48	4.88	**3.75**	±	**1.02**	**3.97**
**295x**	NN-D, Q-D	4.16	4.15	3.98	4.21	**4.13**	±	**0.10**	**4.13**
**297**	NN-D	4.16	4.15	3.98	3.91	**4.05**	±	**0.12**	**4.06**
**300**	Q-D	2.30	4.23	3.71	4.06	**3.58**	±	**0.88**	**3.39**

x or red colour – out of AD

Table S8: Membrane transporters activity predictions with classification models from ChemBench for BTL dataset. ID is the same as in Table S1.

ID	MDR1		BSEP		BCRP		MRP1		MRP2		MRP3		MRP4		MRP5		MCT1		NTCP		ASBT		OCT1		OATP2		PEPT1		BTL*
	I	S	I	S	I	S	I	S	I	S	I	S	I	S	I	S	I	S	I	S	I	S	I	S	I	S	I	S	I
1				■ x					x			x		■		■		■		x		x		■					
2				■		■								■						x									
3	x	■				■		■								■	■ x			x									■
4		■ x				■		■									■			x									■
5	x	■				■		■						■		■	■			x									
6	x	■ x				■		■	x	■							■ x			x				x					■

7	X	X	X			X	X		X	X		X	X			X	X			X		X		X				X	
8	X	X				X	X		X	X		X	X			X	X			X	X			X				X	
9	X	X							X								X			X									
10																				X									
11	X	X	X			X	X		X	X		X	X	X			X			X		X		X					
12		X										X					X			X		X							
13	X	X				X						X	X		X	X	X			X		X							
14																				X									
15	X	X					X													X									
16	X	X															X			X									
17	X	X				X	X		X	X			X				X			X				X					
18	X	X				X	X		X	X		X	X	X		X	X			X		X		X					
19				X					X			X			X					X		X							
20																	X					X							
21		X																	X			X							
22	X	X	X						X	X			X				X			X		X		X					
23	X	X	X			X	X		X	X		X	X	X		X	X		X	X				X				X	
24	X	X				X	X		X	X		X	X	X		X	X			X				X					
25	X	X				X	X		X	X		X	X	X		X	X			X				X					
26	X	X				X	X	X	X	X		X	X	X		X	X			X				X					
27				X								X			X							X							
28																	X												
29																													
30	X	X							X	X			X				X			X		X		X					
31	X	X	X			X			X	X		X	X	X			X		X	X		X		X				X	
32				X					X			X			X					X		X							
33																	X					X							
34		X															X			X		X							
35		X																	X	X									
36												X										X							
37							X			X			X	X	X	X	X							X				X	
38																	X			X									
39																	X			X									
40																	X			X									
41														X															
42													X	X						X	X			X				X	
43	X	X	X			X	X	X	X	X		X	X	X	X	X	X				X	X		X				X	
44						X	X	X					X			X	X			X	X			X			X	X	
45															X	X	X												
46																													
47																													
48																													
49																													
50																													
51																													
52													X	X			X			X		X							
53													X	X			X			X		X							
54													X				X			X		X						X	
55													X	X			X			X		X							
56													X	X			X			X		X							
57													X	X			X			X		X							
58													X	X		X	X			X		X		X				X	
59													X	X	X	X	X			X	X	X		X				X	
60													X	X	X	X	X			X		X		X				X	
61													X	X	X	X	X			X	X	X		X				X	
62														X			X			X								X	
63													X	X			X			X		X							
64														X			X			X		X		X				X	
65																													
66																													
67																													
68														X															
69														X			X												
70																													
71													X	X			X			X									
72													X	X	X	X	X			X	X	X		X				X	
73													X	X			X			X		X							
74														X			X												
75													X	X			X			X		X							
76													X	X			X			X		X							
77													X	X			X			X		X		X					
78														X			X					X							
79													X	X			X			X		X							
80													X	X	X	X	X			X	X	X		X				X	
81														X			X			X		X							
82													X	X		X	X			X		X		X				X	
83													X	X	X		X			X		X							
84													X	X	X		X			X		X		X					

85				x								x			x							x							
86							x					x	x	x	x	x	x			x	x			x				x	x
87						x						x	x	x	x	x	x			x		x		x				x	
88															x	x	x												
89				x								x			x				x			x							
90	x	x		x								x							x			x							
91																				x		x							
92																						x							
93																			x										
94															x	x	x												
95																			x										
96																													
97																													
98															x		x												
99				x																		x							
100	x											x										x							
101															x	x	x												
102				x																									
103	x											x					x		x			x						x	
104													x		x	x	x												
105																													
106																													
107	x					x	x		x			x	x	x	x	x	x		x					x	x			x	
108																	x			x									
109																	x		x										
110																	x												
111															x		x												
112																						x							
113															x	x	x							x					
114																													
115																	x					x							
116	x					x	x					x	x	x		x	x							x	x			x	
117															x	x	x												
118	x		x			x	x	x				x	x	x	x	x	x					x		x				x	
119						x	x					x	x		x	x				x		x						x	
120						x	x					x	x		x	x	x			x		x						x	

x- out of AD, n/a – not applicable, I- inhibition, S- substrate, *new and not available in ChemBench

Table S9: Membrane transporters activity predictions with classification models from ChemBench for phenolic dataset. ID is the same as in Table S2.

ID	MDR1		BSEP		BCRP		MRP1		MRP2		MRP3		MRP4		MRP5		MCT1		NTCP		ASBT		OCT1		OATP2		PEPT1		BTL*
	I	S	I	S	I	S	I	S	I	S	I	S	I	S	I	S	I	S	I	S	I	S	I	S	I	S	I	S	I
1																													
2																													
3																													
4																													
5													x				x			x									
6																													
7													x					x		x				x				x	
8																													
9																													
10													x					x		x				x				x	
11																													
12																													
13																				x									
14																				x									
15																	x			x									
16													x				x			x									
17																						x							
18																													
19																													
20																													
21																		x											
22																		x											
23																		x											
24																						x							
25													x																
26																						x							
27																													
28													x																
29													x									x							
30													x									x							
31																						x							
32													x																

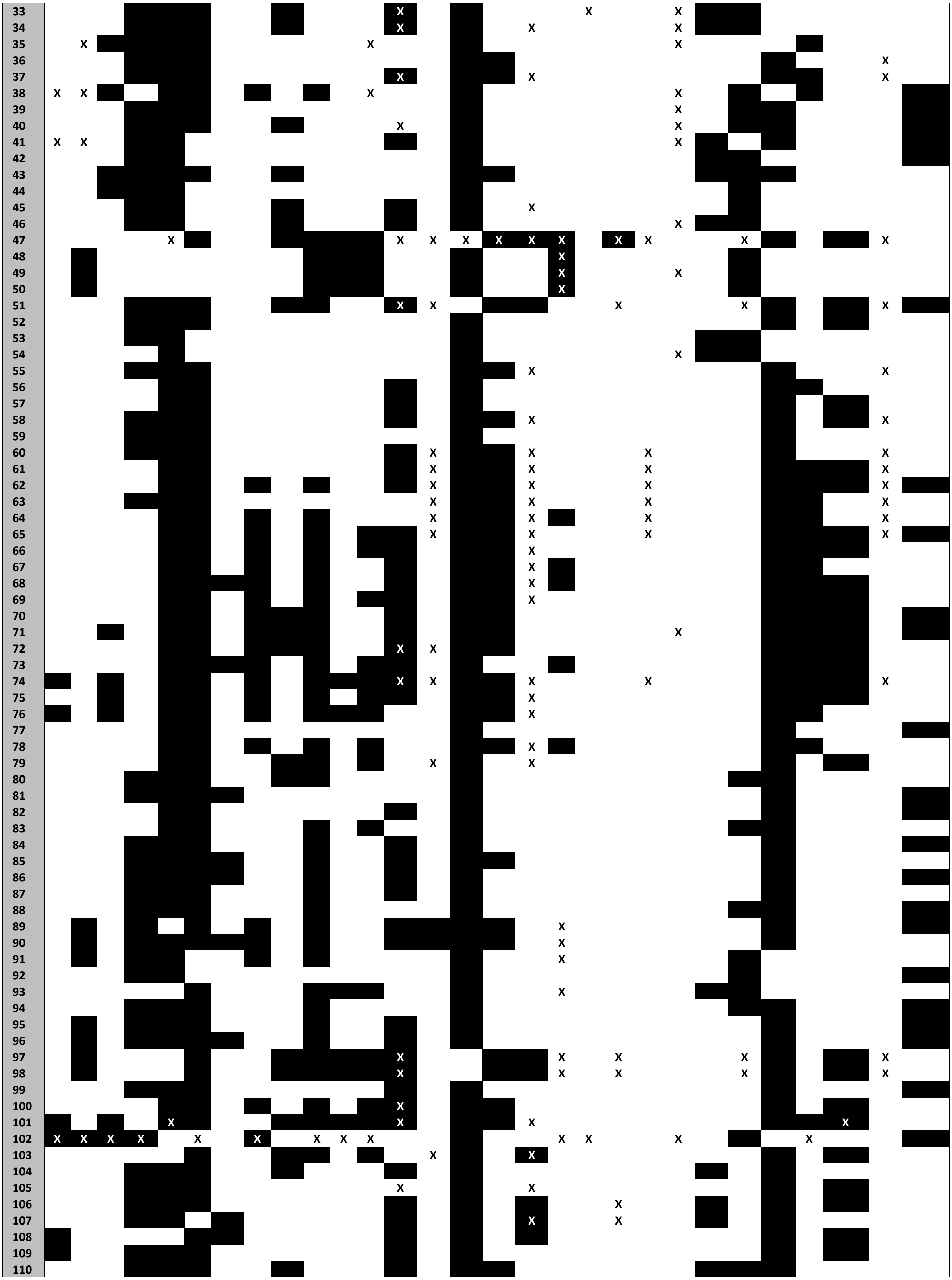
33
34
35
36
37
38
39
40
41
42
43
44
45
46
47
48
49
50
51
52
53
54
55
56
57
58
59
60
61
62
63
64
65
66
67
68
69
70
71
72
73
74
75
76
77
78
79
80
81
82
83
84
85
86
87
88
89
90
91
92
93
94
95
96
97
98
99
100
101
102
103
104
105
106
107
108
109
110

111																													
112																													
113													X							X				X				X	
114																													
115																													
116																		X											
117																													
118																													
119																													
120																													
121																		X											
122														X															
123																													
124																		X											
125													X				X	X											
126													X		X	X		X		X	X			X				X	
127																													
128																		X											
129																													
130																													
131																		X											
132													X	X															
133																						X							
134																						X							
135				X																		X							
136																						X							
137												X										X							
138																						X							
139																						X							
140																						X							
141																													
142													X																
143																													
144	X																												
145	X	X																				X							
146																													
147																													
148																													
149																													
150																													
151																													
152							1																						
153							1							X			X			X									
154							1							X			X			X									
155							1																						
156							1																						
157							1																						
158							1																						
159							n/a		n/a				n/a	n/a	n/a	n/a	n/a			n/a		n/a	n/a	n/a	n/a		n/a	n/a	
160							n/a		n/a				n/a	n/a	n/a	n/a	n/a			n/a		n/a	n/a	n/a	n/a		n/a	n/a	
161							n/a		n/a				n/a	n/a	n/a	n/a	n/a			n/a		n/a	n/a	n/a	n/a		n/a	n/a	
162																	X												
163																													
164																													
165																													
166																													
167																	X			X									
168																													
169																													
170																													
171							n/a		n/a				n/a	n/a	n/a	n/a	n/a			n/a		n/a	n/a	n/a	n/a		n/a	n/a	
172									1																				
173				X						X												X							
174				X																		X							
175				X								X			X							X							
176				X																		X							
177														X			X	X											
178																													
179				X								X										X							
180				X																		X							
181														X								X							
182																		X											
183				X																		X							
184				X																		X							
185				X																									
186				X																									
187				X																									
188														X															

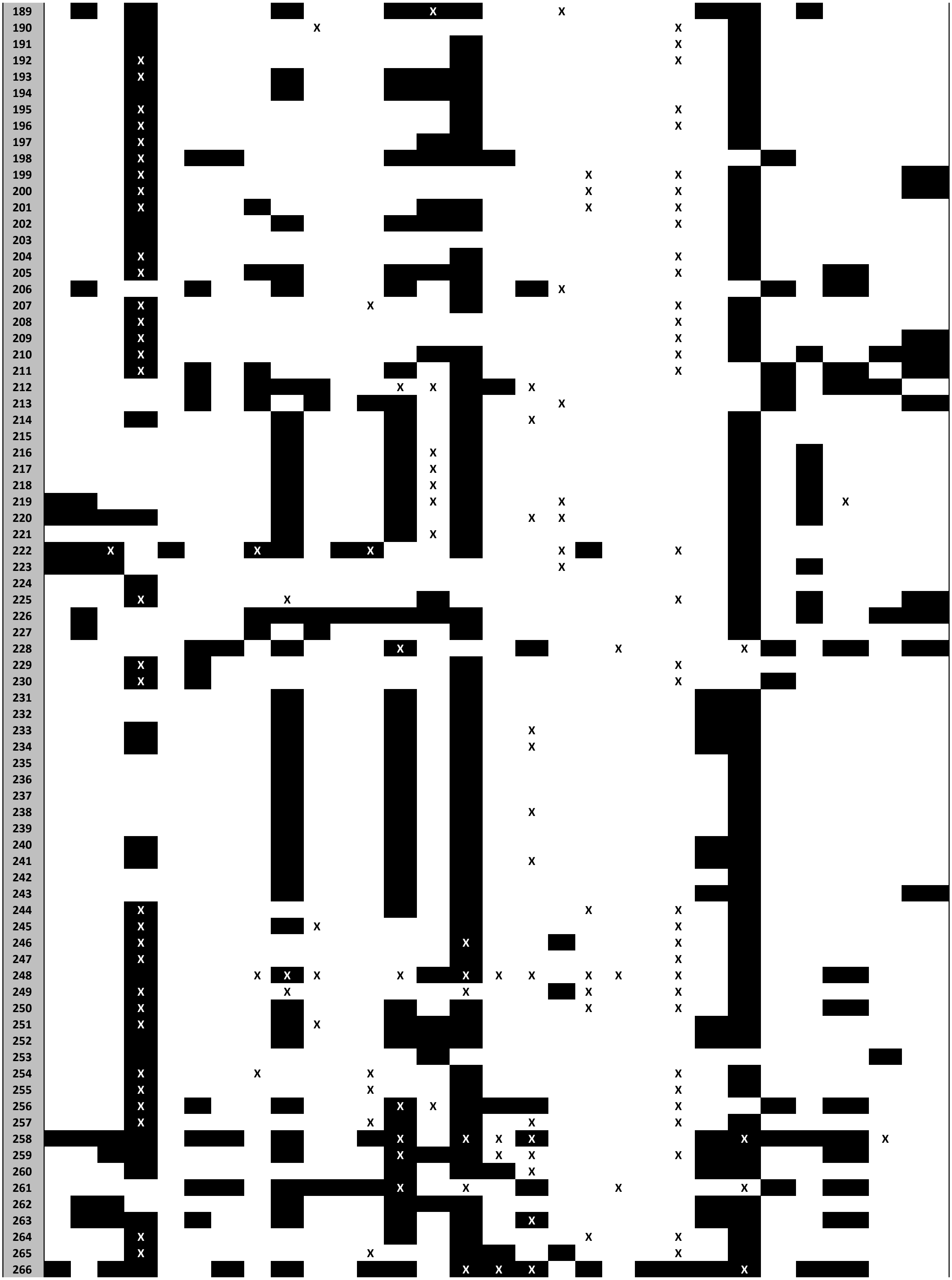
189
190
191
192
193
194
195
196
197
198
199
200
201
202
203
204
205
206
207
208
209
210
211
212
213
214
215
216
217
218
219
220
221
222
223
224
225
226
227
228
229
230
231
232
233
234
235
236
237
238
239
240
241
242
243
244
245
246
247
248
249
250
251
252
253
254
255
256
257
258
259
260
261
262
263
264
265
266

**267**						X		X									X		X			X				X			
**268**				X																		X							
**269**				X																									
**270**																													
**271**																						X							
**272**																						X							
**273**																													
**274**				XX																									
**275**																													
**276**																													
**277**																													
**278**																						X							
**279**	X	X										X										X							
**280**	X																					X							
**281**																	X												
**282**																													
**283**																													
**284**																													
**285**																													
**286**																													
**287**																													
**288**																													
**289**													X			X	X			X									
**290**																													
**291**																	X												
**292**																						X							
**293**													X							X									
**294**																													
**295**																	X												
**296**													X	X						X				X				X	
**297**					X								X	X	X	X	X			X	X			X			X	X	
**298**					X		X						X	X	X	X	X			X	X			X				X	
**299**																						X							
**300**																													

x- out of AD, n/a – not applicable, I- inhibition, S- substrate, *new and not available in ChemBench
